# Supplementary material for: Direct and selective access to amino-poly(phenylene vinylenes)s with switchable properties by dimerizing polymerization of aminoaryl carbenes
Source: Nat Commun. 2021 Jul 2;12:4093. doi: 10.1038/s41467-021-24274-7 (PMC8253808; doi:10.1038/s41467-021-24274-7)
Supplement: Supplementary file 1 — Supplementary information [file 41467_2021_24274_MOESM1_ESM.pdf]

Supplementary Information

Direct and Selective Access to Amino-Poly(Phenylene vinylenes)s with Switchable Properties by Spontaneous Dimerizing Polymerization of Aminoaryl Carbenes

Quentin Sobczak,[1,2] Aravindu Kunche,[1,2] Damien Magis,2 Daiann Sosa Carrizo,[3] Karinne Miqueu,[3] Jean-Marc Sotiropoulos,[3] Eric Cloutet,[1] Cyril Brochon,[1] Yannick Landais,[2] Daniel Taton,[*,1] Joan Vignolle,[*,1]

1. Supplementary Methods

1.1 General Procedures

Materials.
THF and Et_2O were dried over sodium/benzophenone and distilled prior to use. Dichloromethane, chloroform and pentane were dried over CaH_2 and distilled prior to use. Acetonitrile solvent was dried using a MBraun Solvent Purification System (model MB-SPS 800) equipped with alumina drying columns. Benzaldehyde (Alfa Aesar, 98%) was dried over CaH_2, distilled and stored under argon. *n*-Butyllithium solution 11M in hexanes, Lithium bis(trimethylsilyl)amide, Phosphazene (P_4-*t*Bu) 0.8M in hexane and terephthalaldehyde were purchased from Sigma-Aldrich and used without further purification. Diisopropylformamide (Alfa Aesar) and piperidinoformamide (Sigma Aldrich) were stored under argon over activated molecular sieves 4 Å. Trifluoromethanesulfonic acid (TCI), Trifluoromethanesulfonic anhydride (ABCR) and benzaldehyde (Alfa Aesar) were distilled prior to use. Phenyllithium 1.9M in di-n-butyl ether was purchased from Alfa Aesar and used without purification.

Instrumentation.
NMR spectra were recorded on a Bruker Avance 400 (1H, 13C, 19F, 400.2, 100.6 and 376.53 MHz respectively) in appropriate deuterated solvents. Molar masses were determined by size exclusion chromatography (SEC) in THF (1 mL/min) with trichlorobenzene as flow marker, using both refractometric (RI) and UV detectors. Analyses were performed using a three-column TSK gel TOSOH (G4000, G3000, G2000) calibrated with polystyrene standards. HRMS were recorded on various spectrometer: a Waters Q-TOF 2 spectrometer and a GCT premier waters mass spectrometer in the chemical ionization (CI) mode. Melting point were determined by using a Stuart Scientific SMP3 apparatus. Ultraviolet visible spectra were collected on a Thermostated UV/Vis Spectrometer (Agilent Carry 4000). Fluorescence spectra were obtained via Spectrofluorimeter (Jasco FP-8500ST). XRay structures were done on a Rotating Anode Rigaku FRX 3kW with microfocus (Hybrid Dectris Pilatus 200 K pixel detector).

1.2 Synthetic procedures

1.2.1 Precursors synthesis:

Benzylidenediisopropyliminium triflate 1a:

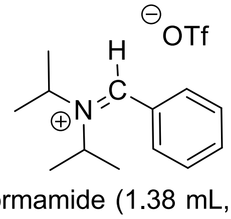

Alder's route: To a stirred solution of diisopropylformamide (1.38 mL, 9.5 mmol) in dry ether (50 mL) cooled at -78°C was added phenyllithium (1.9M, 7.9 mL) dropwise and the resulting mixture was stirred at this temperature for 30 min then at room temperature for 1h. Then, to the reaction mixture cooled at -78°C was added trifluoromethanesulfonic anhydride and the mixture was stirred for 1h at this temperature then for 2h at room temperature. The precipitated solid was filtered under argon and washed with dry ether (3x5 mL) and THF (3x5mL) and dried under vacuum to obtain the compound **1a**, as white crystals (2.1 g, 70%).

Schroth's route: To a stirred solution of benzaldehyde (1 mL, 10 mmol) and 1-(trimethylsilyl) diisopropylamine (1.45 g, 10 mmol) in dry ether (50 mL), TMSOTf (1.8 mL, 10 mmol) was added dropwise at room temperature and the resulting mixture was stirred at the same temperature for 6h under inert atmosphere. The precipitated solid was filtered under inert atmosphere and dried under vacuum to obtain the compound **1a** as white crystals (2.32 g, 70%).

^{1}H NMR (DMSO-*d6*, 400 MHz): δ 1.49 (d, 6H, ^{3}J = 6.6 Hz), 1.54 (d, 6H, ^{3}J = 6.6 Hz), 4.59 (m, 1H), 4.95 (m, 1H), 7.73 (t, 2H, ^{3}J = 6.6 Hz), 7.81 (t, 1H, ^{3}J = 6.6 Hz), 7.85 (d, 2H, ^{3}J = 6.6 Hz), 9.51 (s, 1H);
^{13}C NMR (DMSO-*d6*, 101 MHz): δ 19.16, 23.33, 54.50, 56.98, 120.70 (q, $^{1}J_{C-F}$ = 322.6 Hz), 127.49, 29.38, 131.36, 134.71, 171.31;
^{19}F NMR (DMSO-*d6*, 377 MHz): δ –77.76;

IR (ATR) ν_{max} (cm^{-1}): 2990, 2947, 2885, 2116, 1650, 1600, 1470, 1303, 1275, 1226, 1158, 1033, 972, 832;
HRMS (TOFMS) m/z calcd. For C12H20N^{+} : 190,1596, found 190.1607;
UV/Vis (CH_3CN) λ_{max} = 278 nm;
Mp: 155-160°C.

Benzylidenepiperidineiminium triflate 1b:

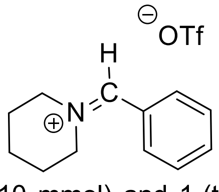

To a stirred solution of benzaldehyde (1.0 mL, 10 mmol) and 1-(trimethylsilyl)piperidine (1.57 g, 10 mmol) in dry ether (50 mL), TMSOTf (1.8 mL, 10 mmol) was added dropwise at room temperature and the resulting mixture was stirred at the same temperature for 6h under inert atmosphere. The precipitated solid was filtered under inert atmosphere and dried under vacuum to obtain the compound **1b** as yellow crystals (2.62 g, 80%).
^{1}H NMR ($CDCl_3$, 400 MHz): δ 1.89 (m, 4H), 2.04 (m, 2H), 4.23 (t, 2H, ^{3}J = 5.6 Hz), 4.29 (t, 2H, ^{3}J = 5.6 Hz), 7.57 (t, 2H, ^{3}J = 7.6 Hz), 7.67 (t, 1H, ^{3}J = 7.6 Hz), 7.78 (t, 2H, ^{3}J = 7.6 Hz), 9.34 (s, 1H);
^{13}C NMR ($CDCl_3$, 101 MHz): δ 22.26, 26.43, 26.58, 52.63, 61.49, 120.67 (q, $^{1}J_{C-F}$ = 322.3 Hz), 126.69, 129.51, 131.19, 134.84, 171.21;
^{19}F NMR ($CDCl_3$, 377 MHz): δ –78.38.
IR (ATR) ν_{max} (cm^{-1}): 2962, 2942, 2873, 2115, 1663, 1600, 1400, 1303, 1275, 1226, 1158, 1032, 972, 832;
HRMS (TOFMS, m/z): Calcd. For $C_{12}H_{16}N^{+}$ 174.1283, found 174,1281;
UV/Vis (CH_3CN) λ_{max} = 272 nm,
Mp: 57-62°C.

9,9'-dihexyl-9H-fluorenylidenepiperidineiminum triflate 1c:

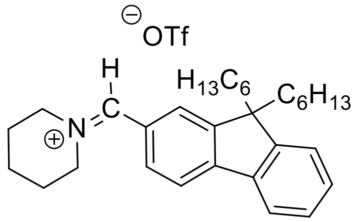

To a stirred solution of fluorene-aldehyde (1.81 g, 5 mmol) and 1-(trimethylsilyl)piperidine (0.79 g, 5 mmol) in dry ether (25 mL), TMSOTf (0.9 mL, 5 mmol) was added dropwise at room temperature and the resulting mixture was stirred at the same temperature for 14h under inert atmosphere. After the solvent was removed under reduced pressure, the resulting mixture was washed with dry cyclohexane (3x20 mL). The resulting solid was filtered under inert atmosphere to obtain the desired compound **1c** as a purple solid (2.25 g, 78%).

^{1}H NMR (THF-d_8, 400 MHz): δ 0.60 (m, 4H), 0.73 (t, 6H, ^{3}J = 7.2 Hz), 1.06 (m, 12H), 1.87 (m, 2H), 1.97 (m, 2H), 2.06 (m, 4H), 2.17 (m, 2H), 4.33 (dt, 4H, ^{3}J = 5.8 Hz), 7.42 (m, 3H), 7.86 (d, 1H, ^{3}J =7.8 Hz), 7.97 (m, 2H), 8.11 (s, 1H), 9.46 (s, 1H);
^{13}C NMR (THF-d_8, 101 MHz): δ 14.30, 23.39, 24.72, 25.31, 27.20, 27.50, 27.75, 30.45, 32.42, 40.64, 53.35, 56.57, 61.95, 121.21, 122.22, 122.29 (q, $^{1}J_{C-F}$ = 321.8 Hz), 124.07, 126.82, 128.17, 128.58, 130.26, 121.82, 140.26, 148.76, 152.51, 153.23, 171.88;
^{19}F NMR (THF-d_8, 377 MHz): δ –79.00.
IR (ATR) ν_{max} (cm^{-1}): 2960, 2932, 2858, 2112, 1654, 1603, 1448, 1273, 1223, 1155.

1,4-dilithiobenzene:

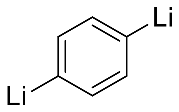

To a stirred solution of dibromobenzene (2.62 g, 11 mmol) in dry ether (100 mL), n-BuLi (4 mL, 44 mmol) was added dropwise at -78°C and the resulting mixture was stirred at room temperature overnight. The precipitated solid was then filtered, washed with dry ether (3x10 mL) under inert

atmosphere and dried under vacuum to obtain the title compound as a light yellow solid (0.890 g, 80%).

Benzylidenebis(diisopropyliminium) bis-triflate 7a:

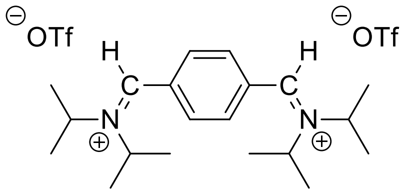

To a stirred solution of 1,4-dilithiobenzene (0.456 g, 5 mmol) in Et_2O at -78°C, diisopropyl formamide (1.32 mL, 9.1 mmol) was added dropwise. The resulting mixture was stirred at -78°C for 30 minutes, followed by 1 hour at room temperature. The mixture was then cooled to -78°C before an ethereal solution of triflic anhydride (*ca*. 1.8 equivalents) was added dropwise with vigorous stirring and let to room temperature overnight. The precipitated solid was filtered, washed with dry ether (3x10 mL), DCM and THF under inert atmosphere and dried under vacuum to obtain the compound **7a** as a light brown solid (1g, 33%). Single crystals were grown from the slow evaporation of a solution of ether in an acetonitrile solution of **7a**.

^{1}H NMR (CD_3CN, 400 MHz): δ 1.54 (d, ^{3}J = 6.6 Hz, 12H), 1.62 (d, ^{3}J = 6.6 Hz, 12H), 4.57 (sept, ^{3}J = 6.6 Hz, 2H), 4.91 (sept, ^{3}J = 6.6 Hz, 2H), 7.96 (s, 4H), 9.26 (s, 2H);
^{13}C NMR (CD_3CN, 400 MHz): 19.90, 24.14, 57.33, 59.64, 122.08 (q, $^{1}J_{C\text{-}F}$ = 321.1 Hz), 131.96, 132.74, 171.85 (t, 1H, J = 12.4 Hz);
^{19}F NMR (CD_3CN, 377 MHz): δ -77.77;
IR (ATR) ν_{max} (cm^{-1}): 3090, 2987, 2885, 2115, 1655, 1467, 1340, 1275, 1225, 1158, 1033, 984, 832;
UV/Vis (CH_3CN) λ_{max}: 287 nm.

Benzylidenebis(piperidineiminium) bis-triflate 7b:

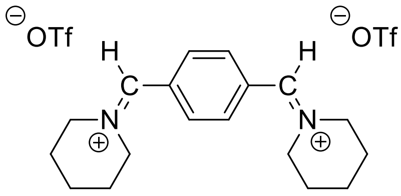

To a stirred solution of terephthalaldehyde (0.27 g, 2 mmol) and 1-(trimethylsilyl) piperidine (0.63 g, 4 mmol) in dry ether (30 mL), TMSOTf (0.72 mL, 4 mmol) was dropwise added at room temperature and the resulting mixture was stirred at the same temperature overnight under inert atmosphere. The precipitated solid was filtered, washed with dry ether (2x25 mL) under inert atmosphere and dried under vacuum to obtain the compound **7b** as a white solid (0.98 g, 86%).

^{1}H NMR (CD_3CN, 400 MHz): δ 1.86 (m, 2H), 1.94 (br, 2H), 2.08 (br, 4H), 4.14 (m, 4H), 7.93 (s, 4H), 9.11 (s, 2H);
^{13}C NMR (CD_3CN, 400 MHz): δ 22.85, 26.18? 27.14, 54.39, 62.99, 68.22, 122.04 (q, $^{1}J_{C\text{-}F}$ = 321.0 Hz), 131.97, 132.37, 170.86 (t, 1H, J = 12.5 Hz);
^{19}F NMR (CD_3CN, 377 MHz): δ –77.76.
IR (ATR) ν_{max} (cm^{-1}): 3092, 2962, 2873, 2115, 1676, 1449, 1275, 1225, 1158, 1033, 972, 832;
UV/Vis (CH_3CN) λ_{max} = 282 nm.

9,9'-dihexyl-9H-fluorenylidenebis(piperidineiminium) bis-triflate 7c:

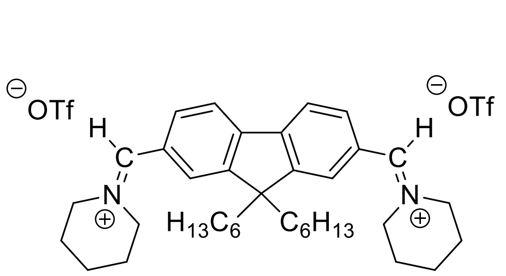

To a stirred solution of 9,9'-dihexyl-9*H*-fluorene-2,7-dicarbaldehyde[1] (1.17 g, 3 mmol) and 1-(trimethylsilyl) piperidine (1.04 g, 6.6 mmol) in dry ether (25 mL), TMSOTf (1.2 mL, 6.6 mmol) was

dropwise added at room temperature and the resulting mixture was stirred at the same temperature overnight under inert atmosphere. The precipitated solid was filtered under inert atmosphere, washed with dry ether (2x25 mL) and dried under vacuum to obtain the compound **7c** as a yellow solid (1.97 g, 80%).

^{1}H NMR (CD_3CN, 400 MHz): δ 0.52-0.60 (m, 4H), 0.73 (t, ^3J = 6.8 Hz, 6H), 1.06 (m, 12H), 1.90 (m, 4H), 2.02 (br, 8H), 2.14 (m, 4H), 4.19 (br, 8H), 7.83 (d, ^3J = 8 Hz, 2H), 7.85 (s, 2H), 8.20 (d, ^3J = 8 Hz, 2H), 9.02 (s, 2H);
^{13}C NMR (CD_3CN, 101 MHz): 14.21, 23.06, 23.14, 24.55, 27.22, 30.06, 32.08, 39.98, 57.34, 122.14 (q, $^1J_{C\text{-}F}$ = 320.6 Hz), 123.43, 127.40K, 128.22, 132.24, 146.21, 153.84, 171.14;
^{19}F NMR (CD_3CN, 377 MHz): δ –79.27;
IR (ATR) ν_{max} (cm^{-1}): 2962, 2934, 2861, 2116, 1656, 1606, 1449, 1275, 1224, 1157, 1032.

Although not mentioned in the main text, other iminiums, such as the thiophene derivative **7d**, the morpholino- **7e** and diethylamino-iminiums **7f** have also been prepared with the aim to investigate the scope of the deprotonation reaction as a mean to access original diaminoalkenes.

Thiophenylidenepiperidine iminium triflate 7d:

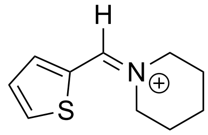

To a stirred solution of thiophenecarboxaldehyde (0,47 mL g, 5 mmol) and 1-(trimethylsilyl)piperidine (0.79 g, 5 mmol) in dry ether (25 mL), TMSOTf (0.9 mL, 5 mmol) was added dropwise at room temperature and the resulting mixture was stirred at the same temperature for 14h under inert atmosphere. After the solvent was removed under reduced pressure, the resulting mixture was washed with dry cyclohexane (3x20 mL). The resulting solid was filtered under inert atmosphere to obtain the desired compound **36** as a violet solid (1.2 g, 73%).

^{1}H NMR (DMSO-d_{6z}, 400 MHz): δ 1.73-1.77 (m, 2H), 1.88-1.95 (m, 4H), 4.04-4.19 (dt, 4H, J = 48.6 and 5.8 Hz), 7.54-7.57 (dd, 1H, J = 5.0 and 3.9 Hz), 8.25-8.27 (dd, 1H, J = 4.0 and 1.4 Hz), 8.65-8.67 (dt, 1H, J = 4.9 Hz and 1.1 Hz), 9.28 (s, 1H).

Benzylidenemorpholine iminium triflate 7e:

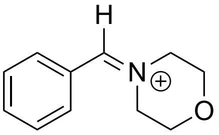

To a stirred solution of benzaldehyde (0,51 mL g, 5 mmol) and 1-(trimethylsilyl)morpholine (0.79 g, 5 mmol) in dry ether (25 mL), TMSOTf (0.9 mL, 5 mmol) was added dropwise at room temperature and the resulting mixture was stirred at the same temperature for 14h under inert atmosphere. After the solvent was removed under reduced pressure, the resulting mixture was washed with dry cyclohexane (3x20 mL). The resulting solid was filtered under inert atmosphere to obtain the desired compound **36** as a white solid (1.14 g, 70%).

Yield: 70%. ^{1}H NMR (DMSO-d_{6z}, 400 MHz): δ 3.88-4.04 (dt, 4H, J = 52.3 and 5.0 Hz), 4.19-4.29 (dt, 4H, J = 31.1 and 5.0 Hz), 7.70-7.74 (m, 2H), 7.80-7.84 (m, 1H), 7.85-7.87 (m, 2H), 9.40 (s, 1H)

Benzylidenediethylamine iminium triflate 7f:

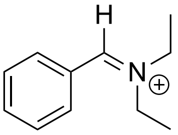

To a stirred solution of benzaldehyde (0,51 mL g, 5 mmol) and 1-(trimethylsilyl)diéthylamine (0.73 g, 5 mmol) in dry ether (25 mL), TMSOTf (0.9 mL, 5 mmol) was added dropwise at room temperature and the resulting mixture was stirred at the same temperature for 14h under inert atmosphere. After the solvent was removed under reduced pressure, the resulting mixture was washed with dry cyclohexane

(3x20 mL). The resulting solid was filtered under inert atmosphere to obtain the desired compound **36** as a white solid (1.15 g, 74%).

^{1}H NMR (DMSO-d_{6z}, 400 MHz): δ 1.44-1.50 (m, 6H), 4.05-4.11 (m, 4H), 7.72-7.76 (m, 2H), 7.82-7.86 (m, 1H), 7.90-7.92 (m, 2H), 9.24 (s, 1H)

1.2.2 Dimerization reaction

1,2-bis(diisopropylamino)stilbene 3a:

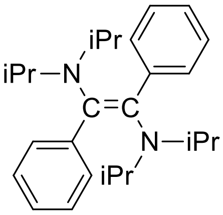

<u>With P4-^{t}Bu base:</u>
To a stirred solution of **1a** (0.1 g, 0.3 mmol) in THF (6 mL) cooled at -78ºC, P_4-^{t}Bu base (0.38 mL, 0.3 mmol, 0.8M in hexanes) was added and the resulting mixture was stirred at this temperature for 1h followed by 24h at room temperature. The solvent was evaporated under reduced pressure and the reaction mixture was diluted with pentane (20 mL) and passed through a short column of basic alumina. The final compound **3a** (0.034 g, 60%) was obtained as a yellow solid. Single crystals were grown from the slow evaporation of a pentane solution of **3a** at -20°C.

<u>With LiHMDS base:</u>
1a (0.1 g, 0.3 mmol) and LiHMDS (50 mg, 0.3 mmol) were dissolved in cold THF (6 mL, -78°C). The resulting mixture was stirred at this temperature for 1h followed by 24h at room temperature. The solvent was evaporated under reduced pressure and the reaction mixture was diluted with pentane (20 mL) and passed through a short column of basic alumina. The final compound **3a** (0.034 g, 60%) was obtained as a yellow solid. Single crystals were grown from the slow evaporation of a pentane solution of **3a** at -20°C.

^{1}H NMR (THF-d_8, 400 MHz): [*E*-isomer] δ 0.77 (d, ^{3}J = 6.5 Hz, 24H), 3.25 (m, 4H), 7.16 (m, 2H), 7.25 (m, 4H), 7.51 (m, 4H) ; [*Z*-isomer] δ 1.20 (d, ^{3}J = 6.8 Hz, 24H), 3.93 (m, 4H), 6.81 (m, 4H), 6.86 (m, 4H), 7.09 (m, 4H);
^{13}C NMR (THF-d_8, 101 MHz): [*E*-isomer] δ 23.48, 52.04, 126.82, 127.97, 132.16, 140.68, 145.59; [*Z*-isomer] δ 24.33, 48.31, 125.52, 127.04, 133.68, 135.06, 144.45;
IR (ATR) ν_{max} (cm^{-1}): 3054, 3018, 2964, 2928, 2866, 1595, 1465, 1441, 1377, 1219, 1193, 1113, 753, 641;
HRMS (TOFMS): Calcd. for $C_{26}H_{38}N_2$ 378.3029, found 378.3030;
UV/Vis ($CHCl_3$) λ_{max} = 245, 388 nm, Mp: 47.22°C.

1,2-bis(piperidine)stilbene 3b:

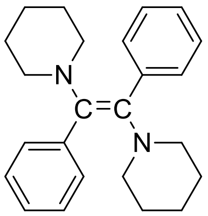

To a stirred solution of **1b** (0.32 g, 1 mmol) in THF (3 mL) cooled at -78ºC, P_4-^{t}Bu base (1.38 mL, 1.1 mmol, 0.8M in hexanes) was added and the resulting mixture was stirred at this temperature for 1h followed by 24h at room temperature. The solvent was evaporated under reduced pressure and the reaction mixture was diluted with pentane (20 mL) and passed through a short column of basic alumina. The final compound **3b** (0.122 g, 70%) was obtained as a yellow solid. Single crystals were grown from the slow evaporation of a pentane solution of **3b** at -20°C.

^{1}H NMR (THF-d_8, 400 MHz): [*E*-isomer] δ 1.36 (s, 12H), 2.38 (br, 8H), 7.16 (m, 2H), 7.31 (m, 8H); [*Z*-isomer] δ 1.60 (m, 12H), 3.00 (m, 8H), 6.93 (m, 6H), 7.06 (m, 4H);
^{13}C NMR (THF-d_8, 101 MHz): [*E*-isomer] δ 25.16, 27.83, 53.56, 127.06, 128.31, 130.33, 137.30, 142.92; [*Z*-isomer] δ 25.46, 27.67, 52.63, 126.14, 127.68, 131.93, 138.54, 141.13;

IR (ATR) v_{max} (cm^{-1}): 3054,3018, 2929, 2850, 2812, 1594, 1489, 1442, 1378, 1228, 1194, 1112, 755, 670;
HRMS (TOFMS): Calcd. for $C_{24}H_{30}N_2$ 346.2403, found 346.2407;
UV/Vis ($CHCl_3$) λ_{max} = 245, 395 nm, Mp: 101°C.

1,2-bis(9,9'-dihexyl-9H-fluorene)-1,2-bis(piperidine)ethene 3c:

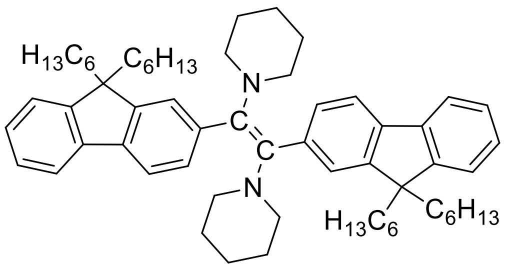

To a stirred solution of **1c** (1.16 g, 2 mmol) in THF (10 mL) cooled at -78ºC, P_4-^{t}Bu base (3 mL, 2.4 mmol, 0.8M in hexanes) was added and the resulting mixture was stirred at this temperature for 1h followed by 24h at room temperature. The solvent was evaporated under reduced pressure and the reaction mixture was diluted with pentane (20 mL) and passed through a short column of basic alumina. The final compound **3c** (0.48 g, 56%) was obtained as an orange solid. Single crystals were grown from the slow diffusion of a pentane solution of **3c** at -20°C.

^{1}H NMR (THF-d_8, 400 MHz): [*E*-isomer] δ 0.57 (m, 20H+4H*), 0.95 (m, 24H+6H*), 1.30 (s, 12H), 1.94 (m, 8H), 2.39 (s, 8H), 7.15 (m, 5H), 7.25 (m, 5H+1H*), 7.59 (m, 4H); [*Z*-isomer] δ 1.52 (m, 12H), 1.74 (m, 8H), 2.98 (br, 8H), 6.99 (m, 2H), 7.05 (m, 4H), 7.39 (m, 2H);
^{13}C NMR (THF-d_8, 101 MHz): [*E*-isomer] δ 14.35, 23.40, 24.74, 25.45, 28.24, 30.70, 32.60, 41.63, 54.00, 55.63, 119.83, 120.20, 123.43, 124.51, 127.45, 127.54, 129.55, 137.44, 140.94, 142.43, 142.57, 151.03, 151.46; [Z-isomer] δ 14.39, 24.64, 25.71, 25.84, 27.96, 30.66, 32.55, 41.42, 52.82, 55.41, 119.29, 120.10, 123.26, 126.27, 127.20, 127.33, 131.22, 138.95, 139.90, 140.82, 142.27, 150.47, 151.50;
IR (ATR) v_{max} (cm^{-1}): 2930, 2848, 1457, 1371, 1122;
HRMS (TOFMS): Calcd. for $C_{62}H_{86}N_2$ 858.6791, found 858.6810;
UV/Vis ($CHCl_3$) λ_{max} = 237, 305, 440 nm.

Deprotonation of 7d, 7e, 7f:

To a stirred solution of **7d-f** (0.1 g, 0.3 mmol) in THF (6 mL) cooled at -78ºC, P_4-^{t}Bu base (0.38 mL, 0.3 mmol, 0.8M in hexanes) was added and the resulting mixture was stirred at this temperature for 1h followed by 24h at room temperature. The solvent was evaporated under reduced pressure and the reaction mixture was extracted with pentane (2 mL). According to ^{1}H NMR in THF-d_8, those reactions were not clean enough to be useful for the investigation of the dimerizing polymerization of related bis-carbenes.

1.2.3 Polymerization reactions

Poly(7,8-bisdiisopropylamino-1,4-phenylenevinylene) 9a:

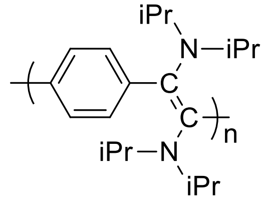

To a stirred solution of **7a** (0.300 g, 0.5 mmol) in THF (2 mL) cooled at -78ºC P_4-tBu base (1.38 mL, 1.1 mmol, 0.8M in hexanes) was added and the resulting mixture was stirred at this temperature for 1h followed by 24h at room temperature. After the solvent was removed under reduced pressure, the mixture was washed with MeOH (10 mL) to remove the phosphazenium salts. The resulting mixture was dissolved in $CHCl_3$ (15 mL) and filtered to remove the insoluble compounds. After evaporation of

the organic solvent, the mixture was purified by precipitation using $CHCl_3$ and MeOH at -10ºC to obtain the desired polymer **9a** as a dark red solid (80 mg, 60%).

1H NMR (THF-d8, 400 MHz): δ 1.05 (m, 24H), 3.32 (m, 4H, E-isomer), 3.91 (m, 4H, Z-isomer), 6.65 (m, 4H), 9.75 (s, 1H), 9.95 (s, 1H).
SEC: $\overline{M_n}$ = 3000 g.mol-1; $\overline{M_w}$ = 6240 g.mol-1; Đ = 2.08
UV/Vis ($CHCl_3$) λ_{max} = 255, 462 nm.
Fluorescence ($CHCl_3$): λ_{ex}= 462 nm, , λ_{em}(liq)=592 nm, λ_{em} (thin film) = 666 nm

Poly(7,8-bispiperidine-1,4-phenylenevinylene) 9b:

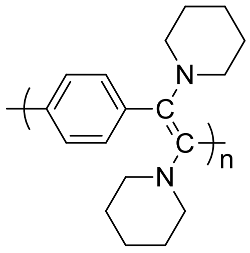

To a stirred solution of **7b** (0.28 g, 0.5 mmol) in THF (2 mL) cooled at -78ºC P_4-^{t}Bu base (1.38 mL, 1.1 mmol, 0.8M in hexanes) was added and the resulting mixture was stirred at this temperature for 1h followed by 24h at room temperature. After the solvent was removed under reduced pressure, the mixture was washed with MeOH (10 mL) to remove the phosphazenium salts. The resulting mixture was dissolved in $CHCl_3$ (15 mL) and filtered to remove the insoluble compounds. After evaporation of the organic solvent, the mixture was purified by precipitation using $CHCl_3$ and MeOH at -10ºC to obtain the desired polymer **9a** as a wine red solid (80 mg, 60%).

^{1}H NMR (THf-*d8*, 400 MHz): δ 1.48 (br, 12 H), 2.41 (br, 4H, *E*-isomer), 3.08 (br, 2H, *Z*-isomer), 3.50 (br, 2H, *Z*-isomer), 7.17 (br, 4H), 9.95 (s, 2H)
SEC: $\overline{M_n}$ = 3900 g.mol^{-1}; $\overline{M_w}$ = 7995 g.mol^{-1}; Đ = 2.05
UV/Vis ($CHCl_3$) λ_{max} = 273, 476 nm
Fluorescence ($CHCl_3$): λ_{ex}= 476 nm, λ_{em}(liq) = 528nm, λ_{em} (thin film) = 685 nm

Poly(7,8-bispiperidine'1,4-(9,9'-dihexylfluorene)vinylene 9c:

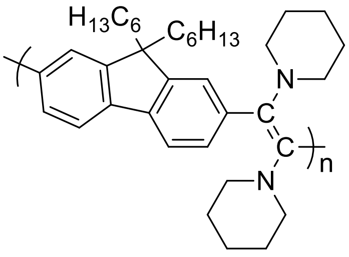

To a stirred solution of **7c** (0.83 g, 1 mmol) in THF (15 mL) cooled at -78ºC P_4-^{t}Bu base (3.0 mL, 2.4 mmol, 0.8M in hexanes) was added and the resulting mixture was stirred at this temperature for 1h followed by 24h at room temperature. After the solvent was removed under reduced pressure, the mixture was washed with MeOH (10 mL) to remove the phosphazenium salts. The resulting mixture was dissolved in $CHCl_3$ (15 mL) and filtered to remove the insoluble compounds. After evaporation of the organic solvent, the mixture was purified by precipitation using $CHCl_3$ and MeOH at -10ºC to obtain the desired polymer **9c** as a reddish-brown solid (425 mg, 81%).

^{1}H NMR (THF-d_8, 400 MHz): 0.78 (m, 10H), 1.12 (m, 12H), 1.44 (br, 12H, *E*-isomer)), 1.68 (br, 12H, *Z*-isomer) 2.03 (m, 4H), 2.52 (br, 8H, *E*-isomer), 3.11 (br, 8H, *Z*-isomer), 7.56 (m, H), 10.02 (s, 2H).
SEC: $\overline{M_n}$ = 5200 g.mol^{-1}; $\overline{M_w}$ = 10660 g.mol^{-1}; Đ = 2.05
UV/Vis ($CHCl_3$) λ_{max} = 240, 483 nm.
Fluorescence ($CHCl_3$): λ_{ex}= 483 nm, λ_{em}(liq) = 561nm, λ_{em} (thin film) = 640 nm.

2. Supplementary discussion

2.1 Optimization of the experimental conditions:

We performed several experiments in order to access higher molecular weight N-PPVs; in particular, influence of the nature of the solvent, base and concentration over the molecular weights was investigated using mainly bis-iminium **7a** as bis-carbene precursor (see Supplementary Table 1). For SEC analysis of N-PPV's from Supplementary Table1, see Supplementary Figure 12 and 13.

Entry	Bis-iminium	Base (2.1 equiv.)	Solvant (concentration)	SEC THF/TEA[a]
1	**7a**	LiHMDS	THF (0.1 M)	M_n = 2030<br>M_w = 3290<br>M_w/M_n = 1.62
2	**7a**	LiHMDS	DMF (0.1 M)	M_n = 1710<br>M_w = 2460<br>M_w/M_n = 1.44
3	**7a**	LiHMDS	Toluene (0.1 M)	M_n = 830<br>M_w = 1680<br>M_w/M_n = 2.04
4	**7a**	P_1-^{t}Bu-$Pyrr_3$	CH_2Cl_2 (0.1 M)	M_n = 1540<br>M_w = 1800<br>M_w/M_n = 1.17
5	**7a**	P_1-^{t}Bu-$Pyrr_3$	CH_2Cl_2 (0.01 M)	M_n = 1539<br>M_w = 1689<br>M_w/M_n = 1.10
6	**7a**	P_1-^{t}Bu-$Pyrr_3$	THF (0.1 M)	M_n = 1560<br>M_w = 1820<br>M_w/M_n = 1.163
7	**7a**	P_1-^{t}Bu-$Pyrr_3$	THF (0.01 M)	M_n = 1400<br>M_w = 1480<br>M_w/M_n = 1.06
8	**7c**	P_1-^{t}Bu-$Pyrr_3$	THF (0.01 M)	M_n = 3580<br>M_w = 5870<br>M_w/M_n = 1.64
9	**7b**	P_1-^{t}Bu-$Pyrr_3$	THF (0.01 M)	M_n = 1830<br>M_w = 2860<br>M_w/M_n = 1.56
10[b]	**7a**	LiHMDS<br>(1 M THF)<br>0.32 mL/h<br>3 h 20 min	THF (0.01 M)	M_n = 1810<br>M_w = 3380<br>M_w/M_n = 1.87
11[c]	**7a**	LiHMDS<br>(1 M THF)<br>32 mL/h<br>2 min	THF (0.01 M)	M_n = 1840<br>M_w = 3490<br>M_w/M_n = 1.89

[a] 10% of Et3N was added to the THF eluent because of the interaction between N-PPV's and the columns.
[b] A slow addition of base was performed with a syringe pump at a rate of 0.32 mL/h (over 3h20).
[c] For comparison, the base was added at a rate 100 times faster (32 mL/h) over 2 min.

Supplementary Table 1. Optimization of the polymerization reaction

The following procedure (optimized) was used for all reactions performed in Supplementary Table1:

Bis-iminium triflate salt (0.5 mmol, 1 equiv.) was charged in a Schlenk flask under an argon atmosphere of a glovebox and suspended in a selected distilled solvent (partial soluble in THF, DCM, toluene but completely soluble in DMF) at 25 °C. This solution was cooled to – 90 °C using ethanol/liquid nitrogen bath. A solution of base (1.05 mmol, 2.10 equiv.) in the appropriate solvent was added dropwise at -90 °C under strong stirring. The mixture was allowed to very slowly warm to room temperature by letting the cold bath warm over 16 h. The obtained dark red solution was then quenched by addition of methanol and stirred for 10 min at room temperature. The solvent was evaporated under vacuum and the red solid transferred to a Soxhlet extractor equipped with a glass fibre cartridge. This crude product was washed by Soxhlet extraction with methanol during 16 h to remove any salts and low molecular weight oligomers. The remaining polymer in the cartridge was

subsequently extracted with chloroform until total decolouration of the extracting solution. The final polymer was obtained free from salts after evaporation of chloroform under vacuum (70-99 % yield).

For instance, $NiPr_2$-PPV **9a** was synthesized following this general procedure:

Benzylidenebis(diisopropyliminium) triflate (0.30 g, 0.5 mmol) partially soluble in THF (50 mL) was deprotonated with a solution of tert-Butylimino-tri(pyrrolidino)phosphorane (0.32 mL, 1.05 mmol) in 2 mL THF to avoid freezing at -90 °C. $NiPr_2$-PPV polymer was obtained as a dark red solid upon purification (105 mg, 70 %).

2.2 Protonation reactions

2.2.1 Protonation of 9b:

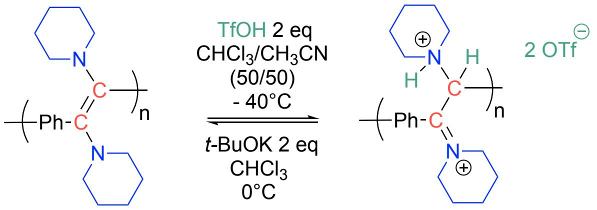

9b, 9b' **10b**

To a deep red solution of polymer **9b** (0,134 g, 0.50 mmol) in $CH_3CN/CHCl_3$ (50/50) cooled at -40°C, TfOH (184.2 µL, 2.2 mmol) was added dropwise. The resulting mixture was warm to room temperature overnight, leading to a yellow solution. Finally, after evaporation of the solvents under vacuum **10b** was isolated in 99% yield (0.245 g). According to UV/visible spectroscopy, the characteristic absorption (λ_{max} = 476 nm) of **9b** was lost and was replaced by an absorption (λ_{max} = 254 nm) in the spectrum of **10b**, suggesting a loss of the conjugation. Note that this reaction is reversible since addition of t-BuOK to $CH_3CN/CHCl_3$ (50/50) solution of **10b** yielded back to **9b** (**9b'**), where the λ_{max} (462 nm for **9b'**) was restored.
A similar procedure was followed for **9a** and **9c**; the same behavior was observed during the protonation of **9c** into **10c**.

2.2.2 Protonation of 9a:

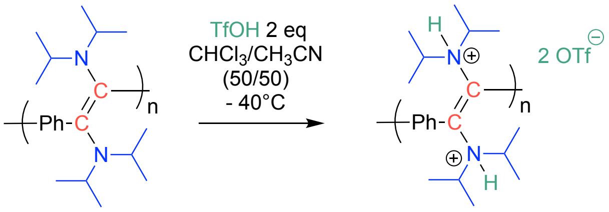

9a **10a**

A completely different scenario occurred during the protonation of **9a**. In this case, the characteristic absorption (λ_{max} = 462nm) of **9a** was blue-shifted down to 352 nm in **10a**, suggesting an alteration of the conjugation in **10a** (the solution was orange-brownish).
To gain some insight into the different behavior of **9a** and **9b,c**, protonation of the corresponding molecular dimers **3a** and **3b** (as representative of piperidine-substituted dimers **3b,c**) was carried out with 2 eq. of TfOH (see below). Hence, while both nitrogen atoms are protonated and the C=C bond is preserved in the case of **3a**, protonation of **3b** occurs at one nitrogen atom and at the enamine carbon, ultimately leading to a dicationic compound without C=C bond. By analogy, we may hypothesize that protonation of N-PPV's **9a** and **9b** led respectively to conjugated **10a** and non-conjugated **10b**, in agreement with

respectively, the disappearance and a blue-shift of the λ_{max} in the UV/vis spectrum of **9a** and **9b** upon protonation.

2.2.3 Protonation of 3a and 3b:

Procedure: 2 eq. of TfOH (0.26mL, 3 mmol) were added at -78°C to a stirred DCM solution (1mL) of **3-E/Z** (1.5 mmol). The reaction was stirred at this temperature for 1h and the solution was slowly warmed to RT overnight. After evaporation of the solvent under vacuum, the remaining solid was washed with Et_2O and dried under vacuum to afford a mixture of mono protonated **Z-3-HOTf** and di-protonated **E-3-(HOTf)$_2$** isomer. Further washing with THF (3x1mL) allowed to selectively removing mono-cationic **Z-3-HOTf** isomers. Recrystallization of **E-3a-(HOTf)$_2$** and **E-3b-(HOTf)$_2$** from THF/CH_3CN mixture afforded single crystals suitable for diffraction analysis (see section Crystallographic data, section 8).

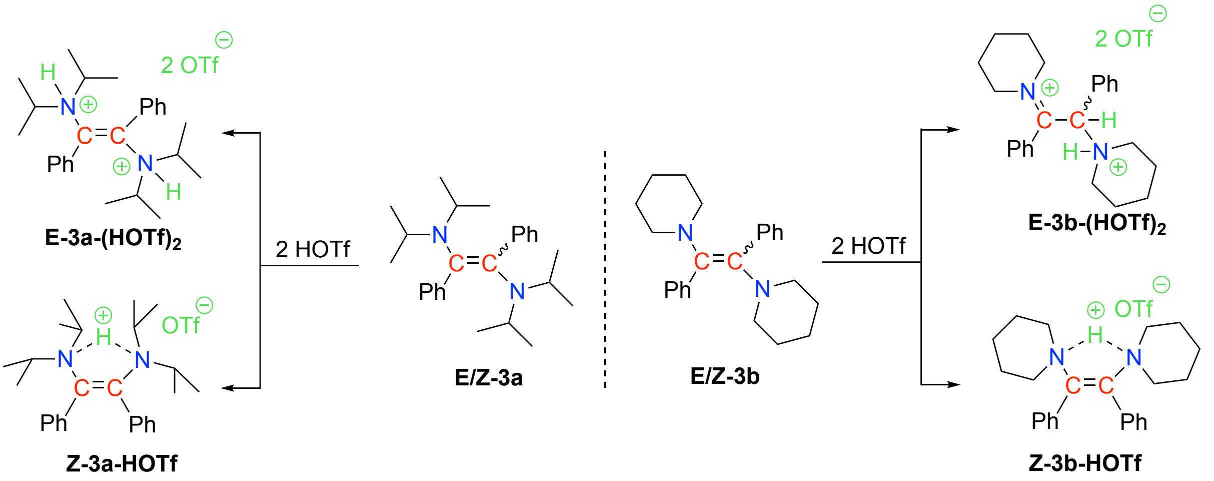

2.3 Addition reaction

Benzylidenediisopropyliminium-2-dithiocarboxylate 4a:

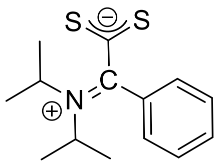

To a stirred solution of **1a** (0.146 g, 0.45 mmol) and S_8 (1.16 g, 4.5 mmol) in THF (5 mL) cooled at -78ºC, LiHMDS (0.5 mL, 0.5 mmol, 1.0 M in THF) was added and the resulting mixture was stirred at this temperature for 1h then warmed to room temperature overnight. Evaporation of the solvent followed by silica-gel column chromatography (n-hexane/ethyl acetate) gave the compound **4a** as a yellow powder (0.069 g, 70 %). Single crystals were grown from the slow diffusion of a solution of ether in a THF solution of **4a**.

^{1}H NMR (THF-d_8, 400 MHz): δ 1.32 (m, 2H), 1.44 (m, 1H), 1.52 (m, 3H), 3.50 (t, ^3J = 5.6 Hz, 2H), 4.31 (t, ^3J = 5.6 Hz, 2H), 7.25 (m, 5H).
^{13}C NMR ($CDCl_3$, 101 MHz): δ 199.5, 143.3, 128.3, 128.2, 125.3, 53.1, 50.5, 26.8, 25.4, 24.1.

2.4 Film forming properties of N-PPV's:

The film forming properties of N-PPV's 9 were investigated with **9c** as representative example. Gratifyingly, a film of **9c** could be prepared from a $CHCl_3$ diluted solution (15 mg/mL) and spin-coating (speed: 2000 rpm) on a glass substrate previously treated by UV/O_3 during 30 minutes. Photos of the film of **9c** are shown below (left normal size from *Canon camera EOS 700d*, right optical image from a *Nikon Eclipse Ti-E microscope*):

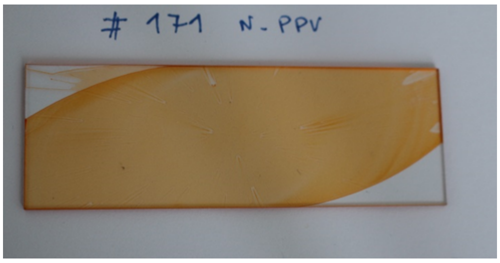

Supplementary Figure 1. Photo of the film of **9c**

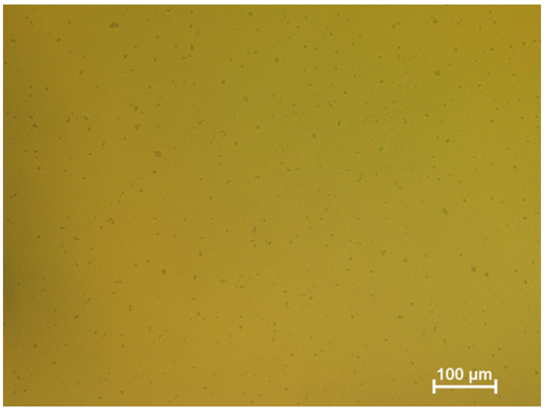

Supplementary Figure 2. Optical image of the film of **9c**

The film thickness and the surface topography were obtained by Surface Force Microscopy in tapping mode (SFM, Bruker Dimension FastScan). Silicon cantilevers (FastScan A) with a tip radius of around 5 nm were used. The resonance frequency of the cantilevers was about 1400 kHz.

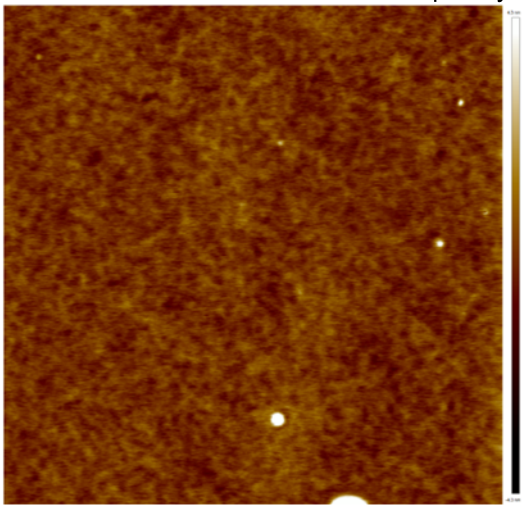

Supplementary Figure 3. 10x10 µm SFM topographic image illustrating that a homogeneous film was formed

Thickness of the film was evaluated as 85 nm (see right graph below showing surface's profile) by SFM via a scratch made on the film (see left image below):

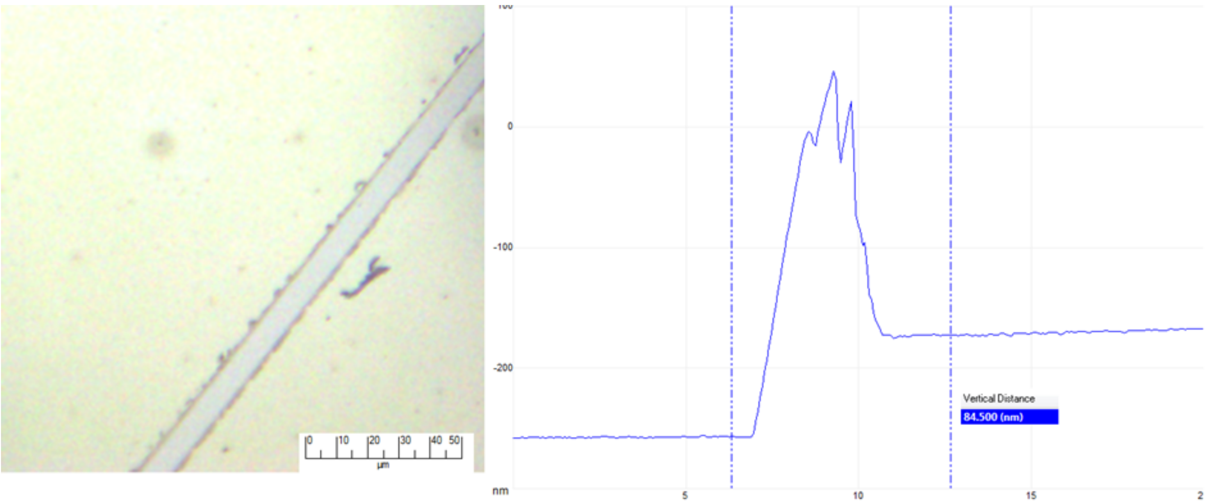

Supplementary Figure 4. Determination of the thickness of the film via a scratch made on the film of **9c** (left) and surface profile determined by SFM (right)

2.5 NMR spectra

2.5.1 Characterization of Dimers 3a,b,c and relative N-PPV's 9a,b,c

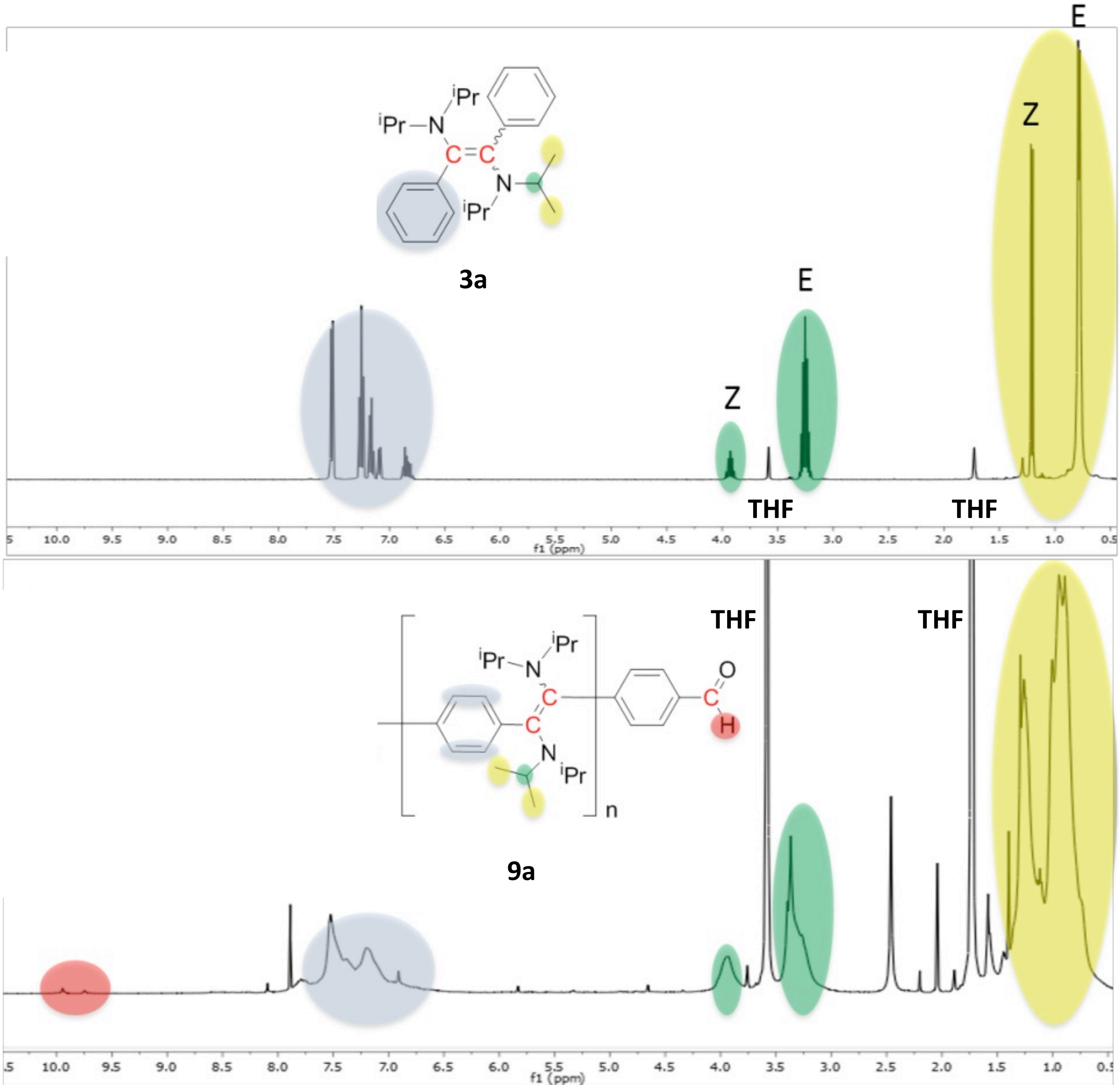

Supplementary Figure 5. ^{1}H NMR of dimer **3a** and its corresponding polymer **9a** in THF-d8.

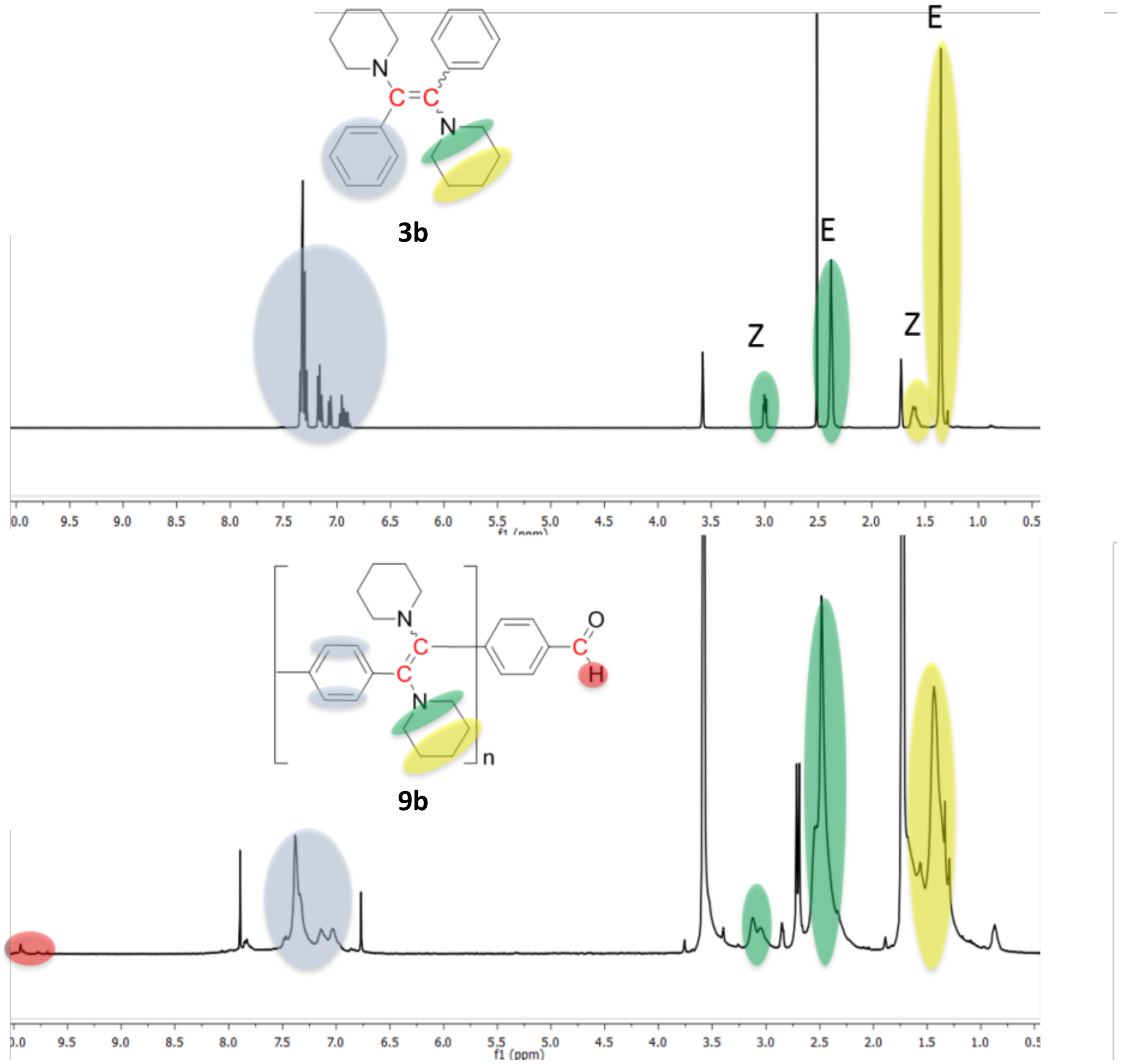

Supplementary Figure 6. ${}^{1}H$ NMR of dimer **3b** and its corresponding polymer **9b** in THFd8.

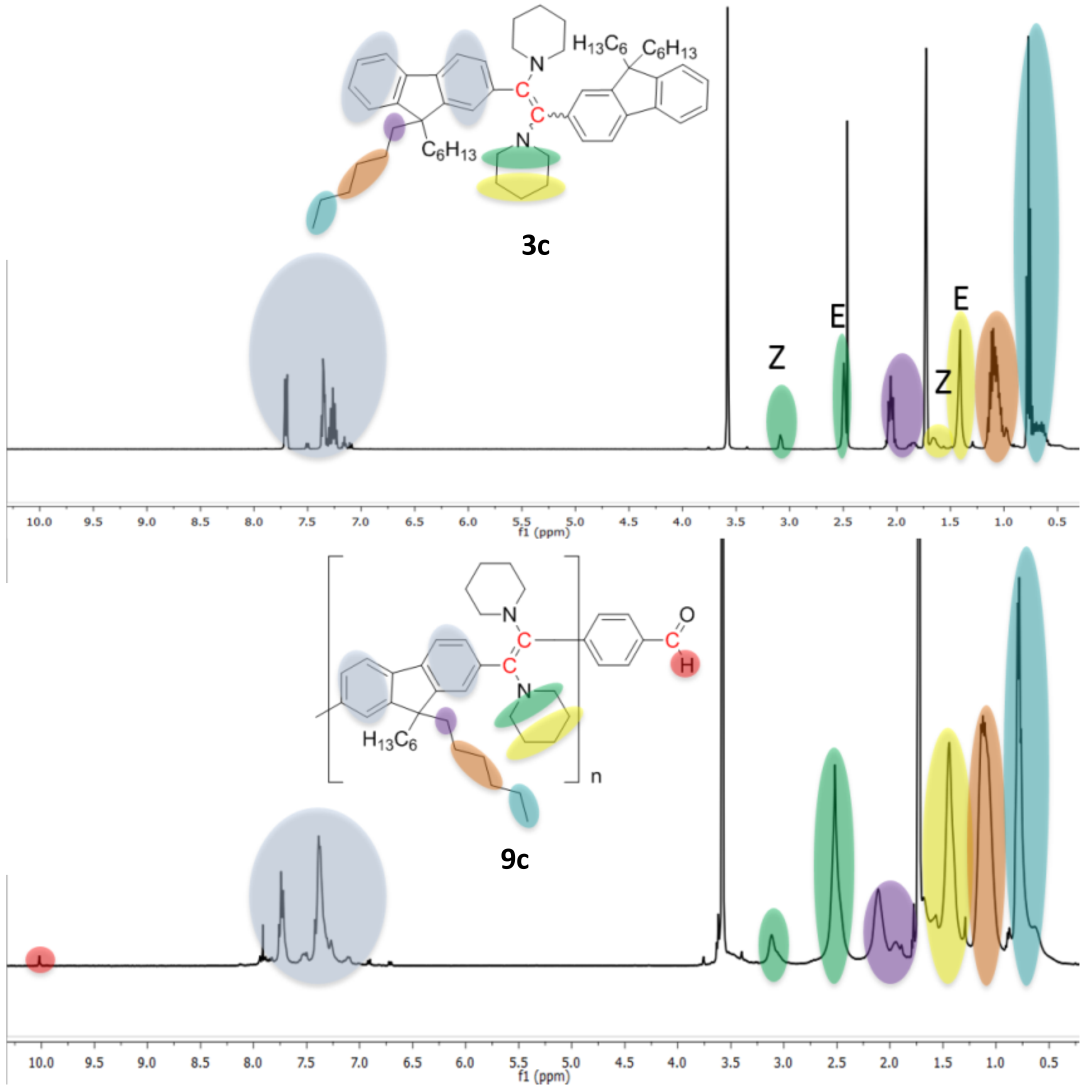

Supplementary Figure 7. ^{1}H NMR of dimer **3c** and its corresponding polymer **9c** in THF-d8.

In order to calculate the NMR DPn, the aldehyde peak was first integrated to a value of two, corresponding to the two aldehydes end-chain functions in the polymer chain. Then, the calculus was done as followed: addition of the protons corresponding of both green peaks, then, the result is divided by the number of protons corresponding of the green peaks in the repeating unit. For example for **9c**, the values of both green peaks is equal to 366 protons divided by 8 protons in the repeating unit gave a DPn of 45.

2.5.2 Investigation of the stability of 3a by variable temperature (VT) NMR analysis

To gain some insight into the stability of N-PPVs **9**, we investigated the stability of the corresponding diaminoalkenes **3** towards their potential dissociation into the corresponding amino(aryl)carbenes. For this purpose, **3a** was selected as representative example because *i*-Pr substituents give characteristic signals in ^{1}H NMR (CH gives a septet and CH_3 gives a doublet); moreover, the chemical shift of CH*i*-Pr is very sensitive to the environment around the nitrogen atom (*i.e.* its hybridization). Thus, VT NMR was performed over 20-90 °C temperature range, using toluene-d8 as solvent. A superimposition of the different spectra is given in figure X. As can be seen, **3a** remains perfectly stable over the whole

range of temperature as no signals corresponding to the amino(aryl)carbene nor signals corresponding to rearrangement/decomposition products could be observed.

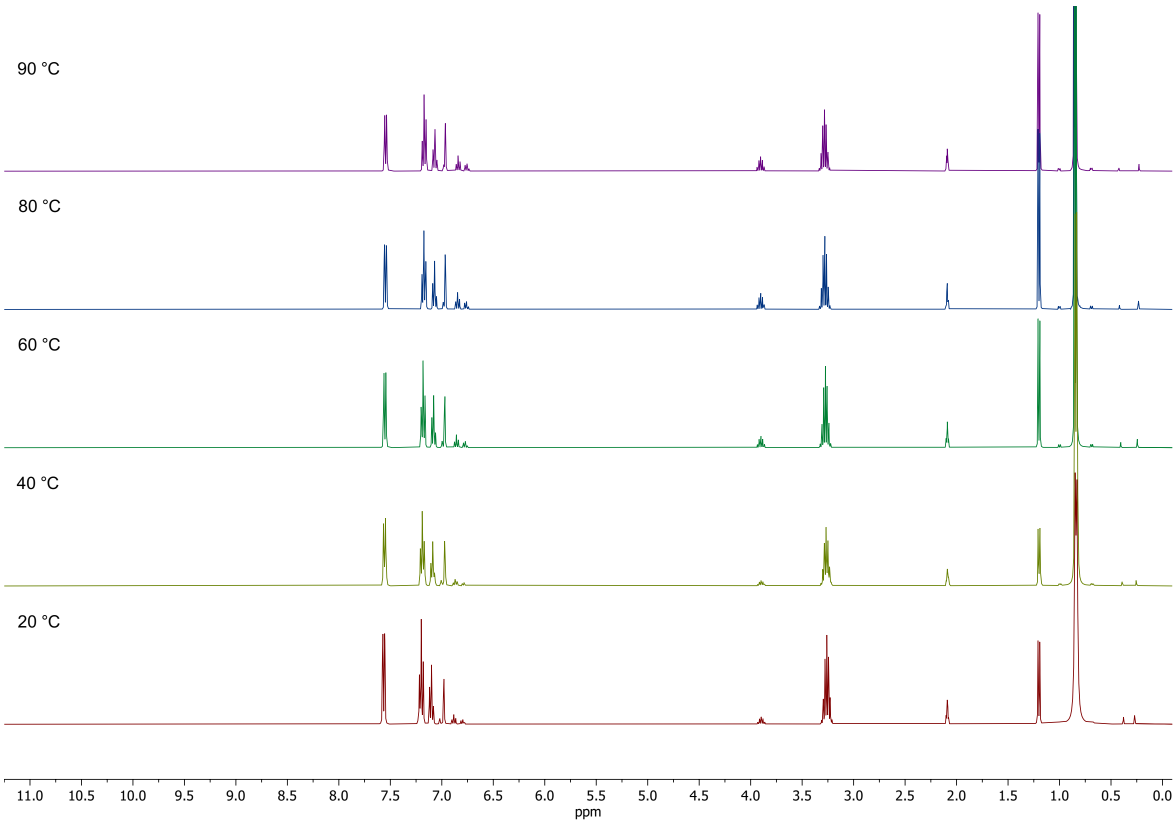

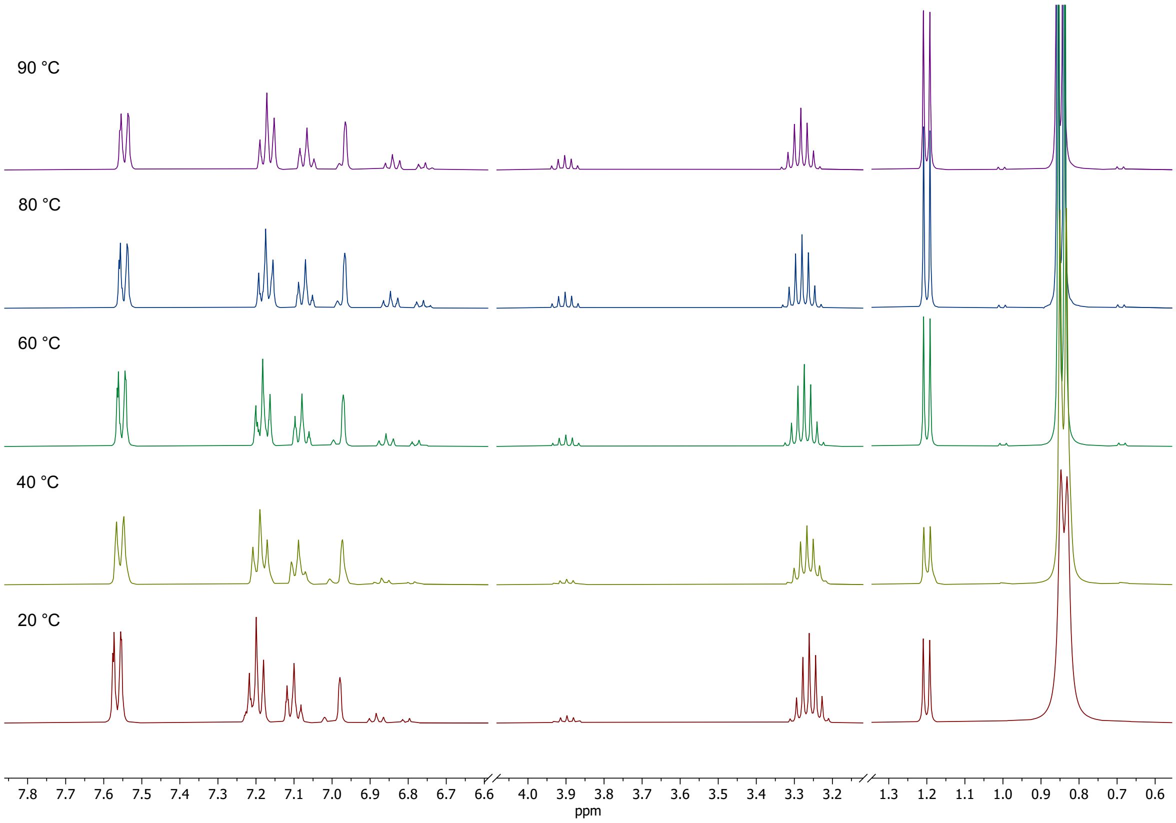

Supplementary Figure 8. VT ^{1}H NMR of dimer **3a** in Toluene-d8 (full spectrum and partial spectrum with enlarged selected areas).

2.6 Size exclusion chromatography spectra

The calibration PS standard was used for the SEC experiments.

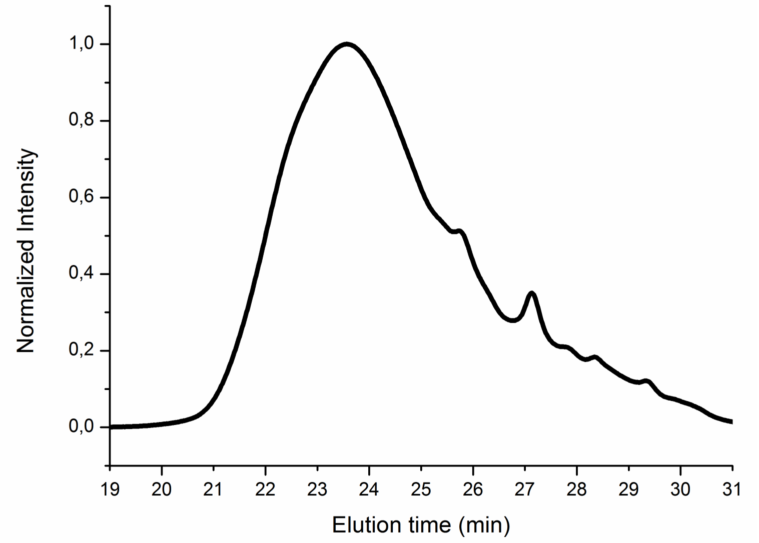

Supplementary Figure 9. SEC spectrum of compound **9a** in THF Mn = 3000 g.mol^{-1} and Đ = 2.08.

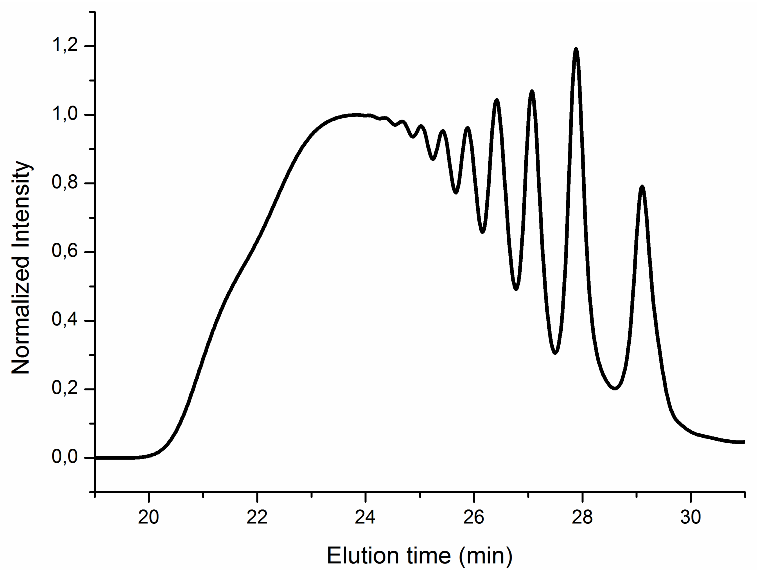

Supplementary Figure 10. SEC spectra of compound **9b** in THF Mn = 3900 g.mol^{-1} and Đ = 2.05.

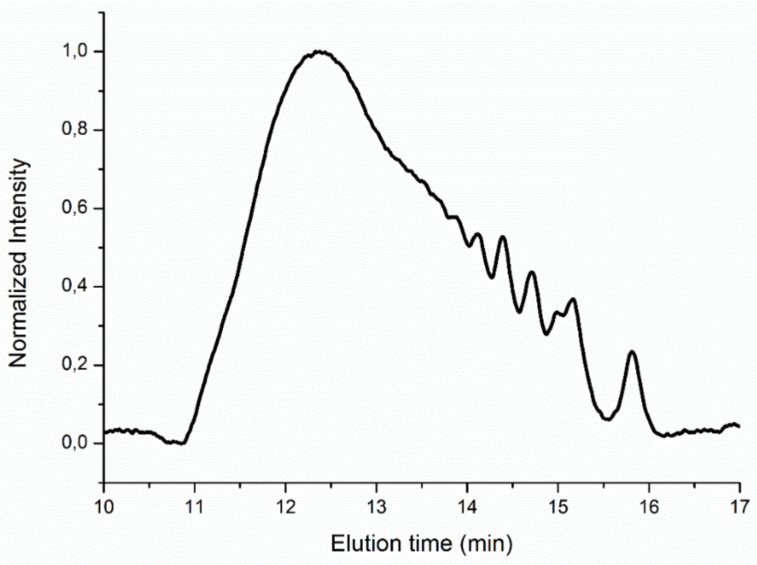

Supplementary Figure 11. SEC spectra of compounds **9c** in THF Mn = 5200 g.mol^{-1} and Đ=2.60.

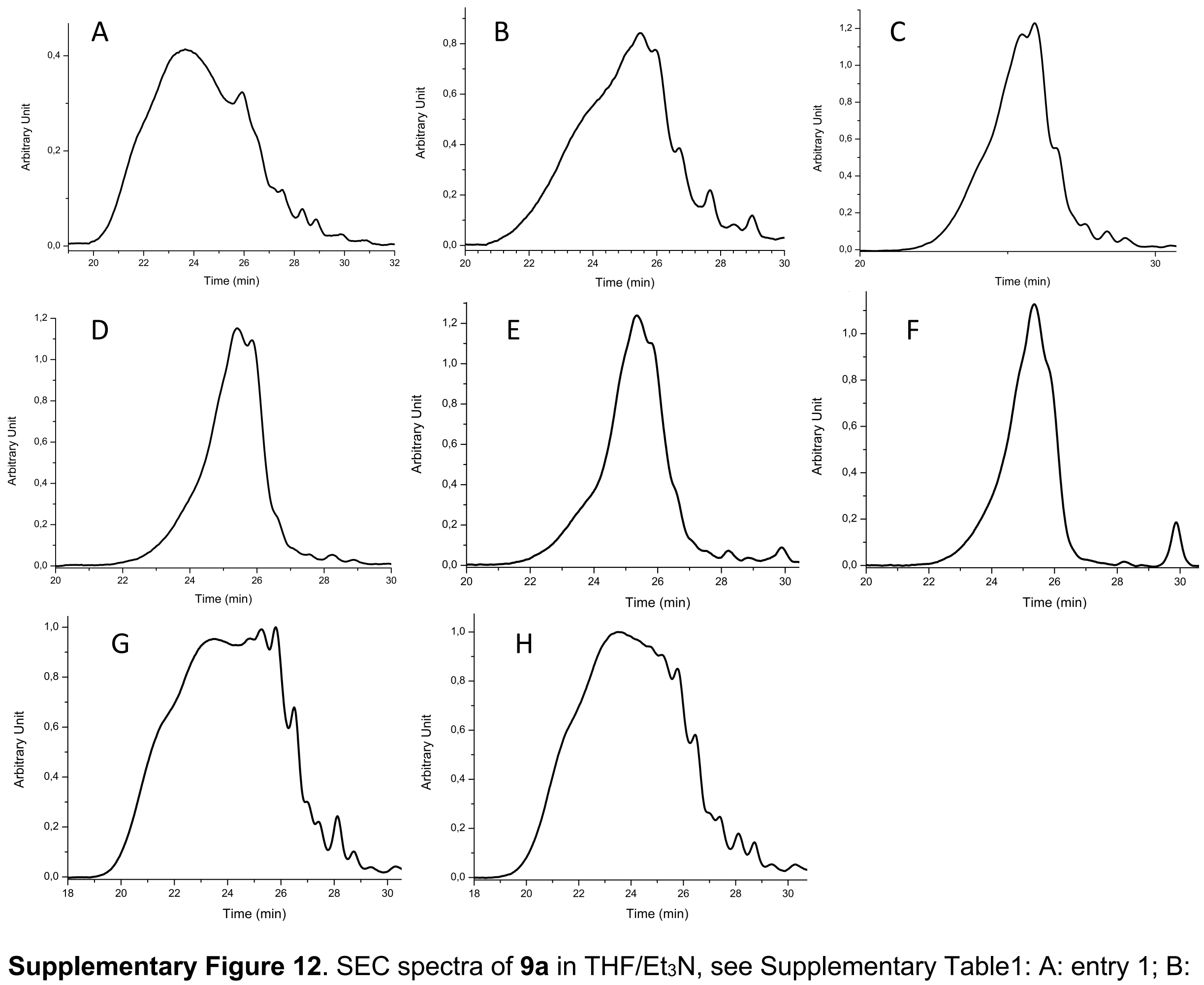

Supplementary Figure 12. SEC spectra of **9a** in THF/Et_3N, see Supplementary Table1: A: entry 1; B: entry 2; C: entry 6; D: entry 7; E: entry 8; F: entry 9; G: entry 4; H: entry 5.

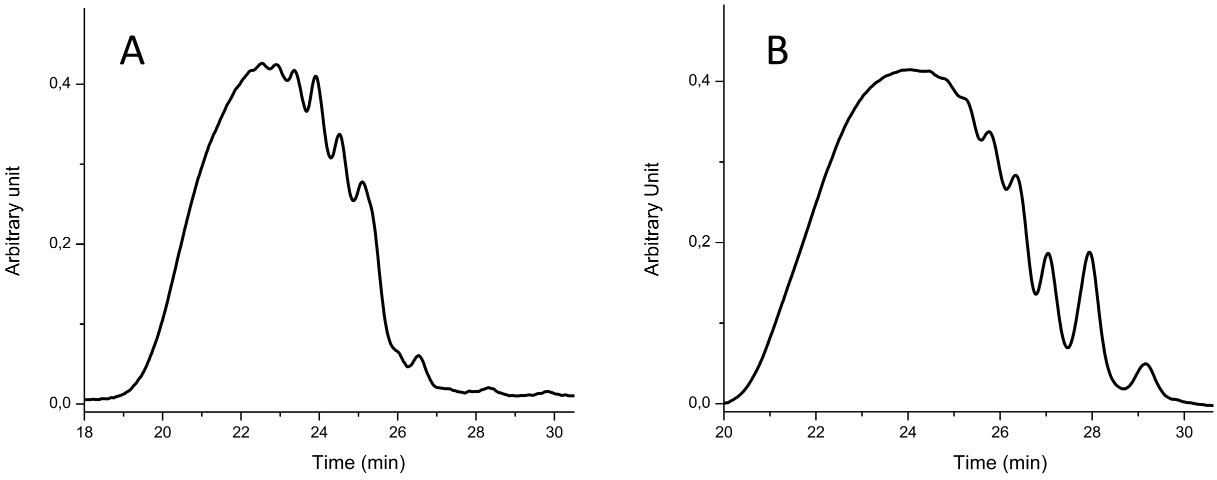

Supplementary Figure 13. SEC spectra in THF/Et_3N of **9b** (B: entry 11) and **9c** (A: entry 10)**,** see Supplementary Table1.

2.7 Absorption and emission spectra of N-PPV's 9

2.7.1 In solution

For absorption, see UV/visible spectra of **9a,b,c** in the main text (figure 4).

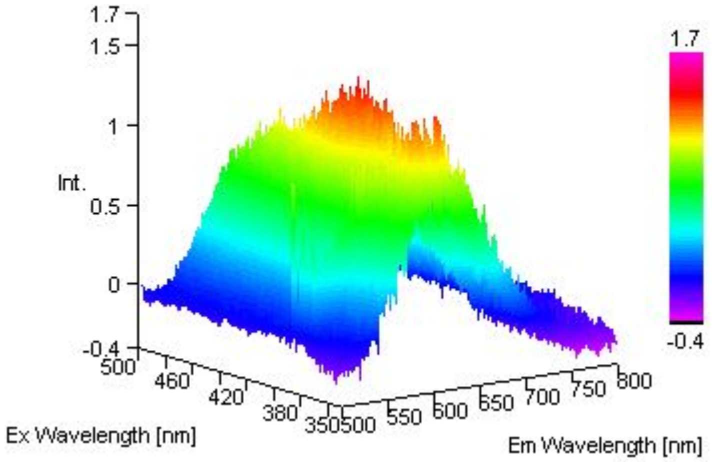

Supplementary Figure 14. Emission spectra of compounds **9a** in $CHCl_3$ solution.

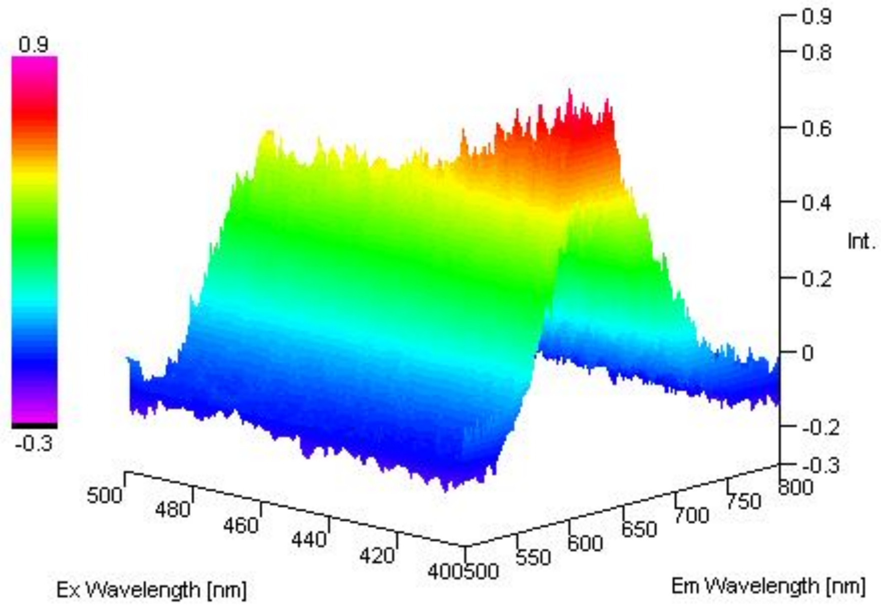

Supplementary Figure 15. Emission spectra of compounds **9b** in $CHCl_3$ solution.

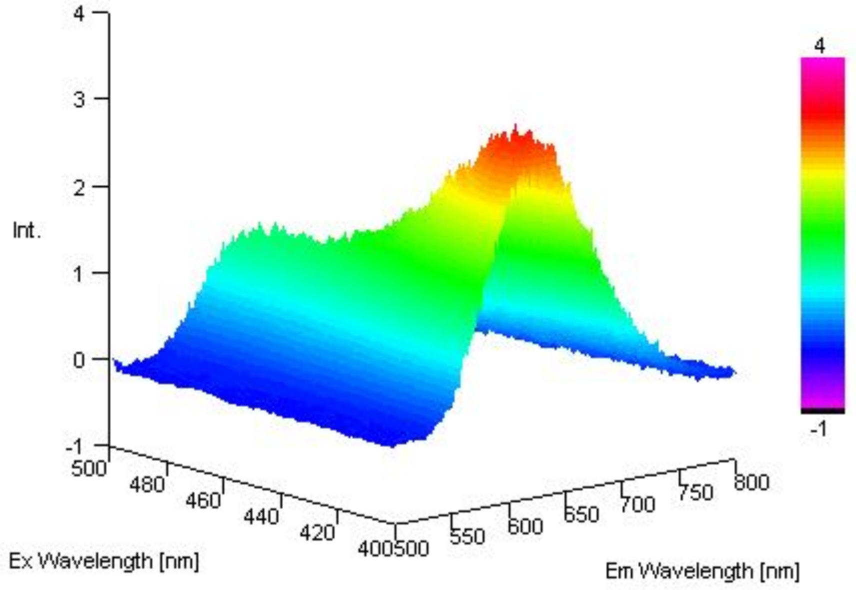

Supplementary Figure 16. Emission spectra of compounds **9c** in $CHCl_3$ solution.

2.7.2 In thin film

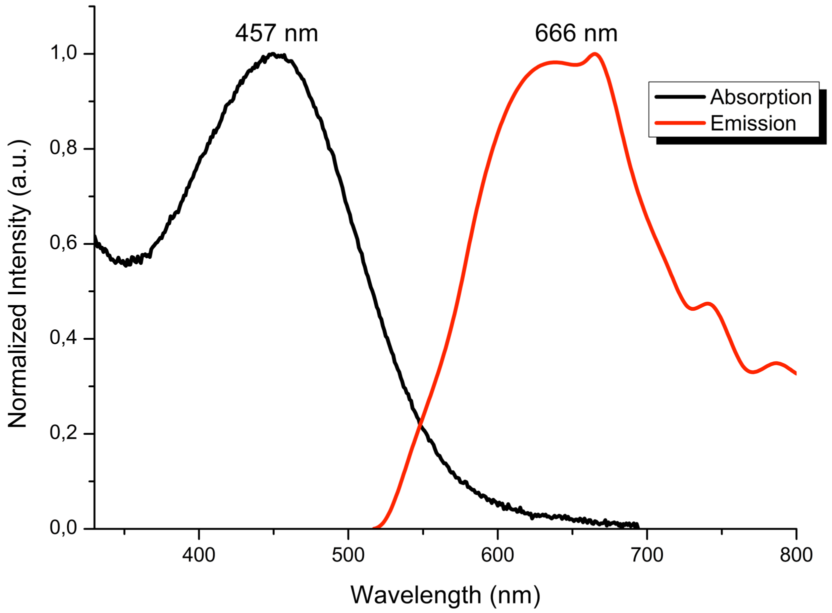

Supplementary Figure 17. UV/Vis and emission spectra of compounds **9a** in thin film.

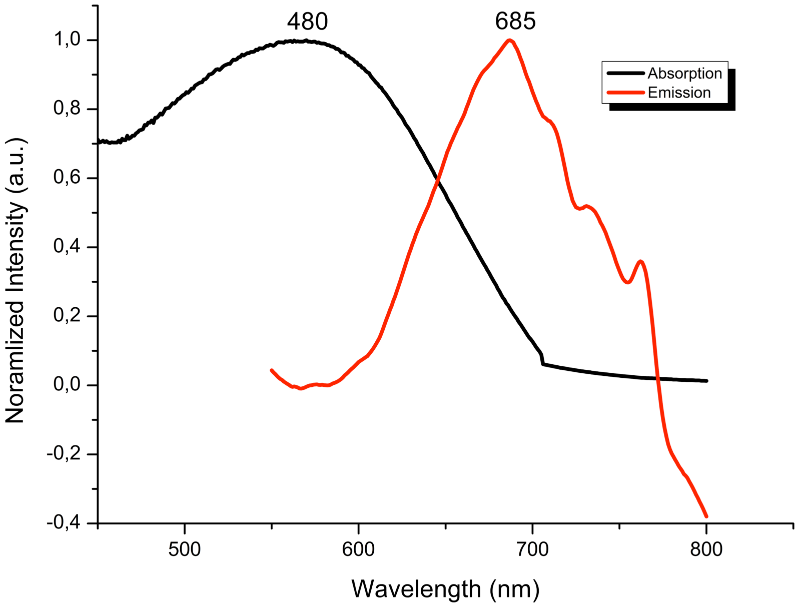

Supplementary Figure 18. UV/Vis and emission spectra of compounds **9b** in thin film.

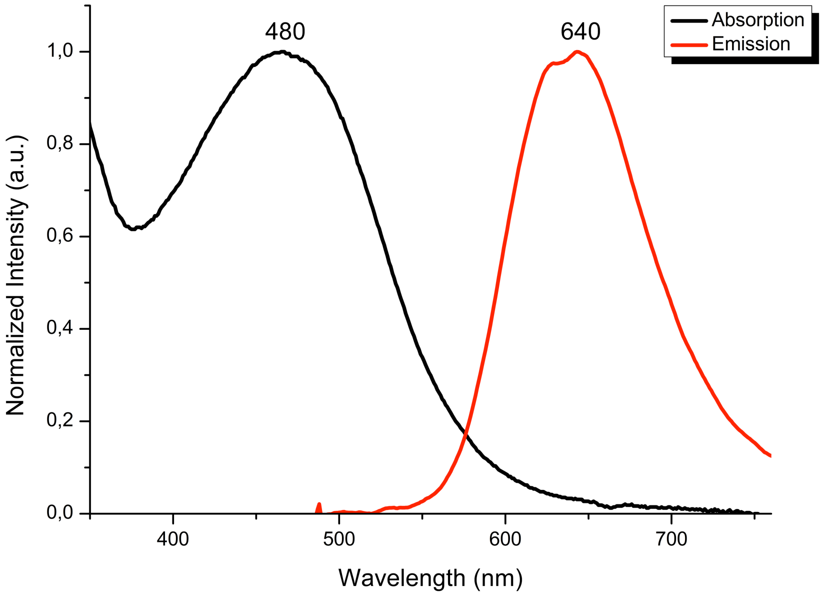

Supplementary Figure 19. UV/Vis and emission spectra of compounds **9c** in thin film.

2.8 MALDI analysis

a)

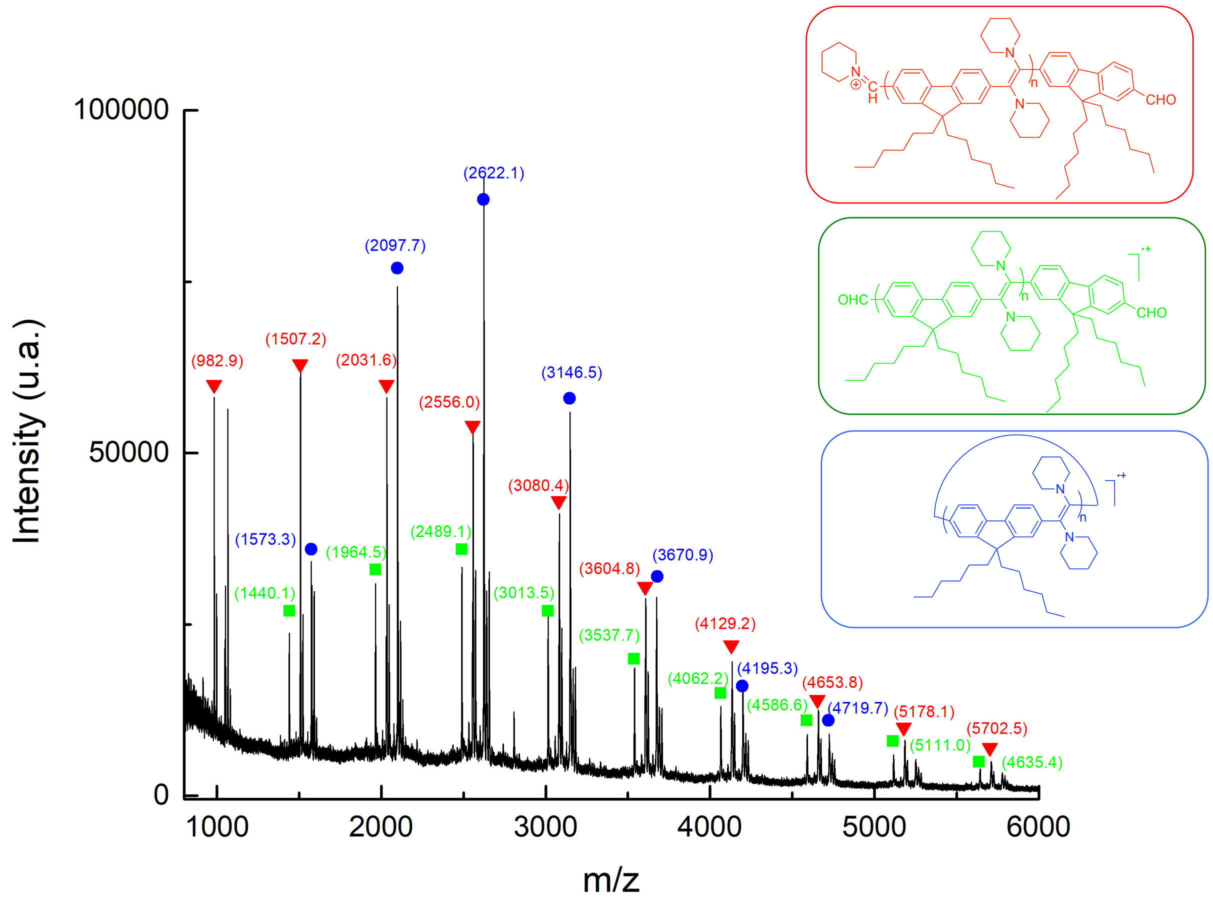

b)

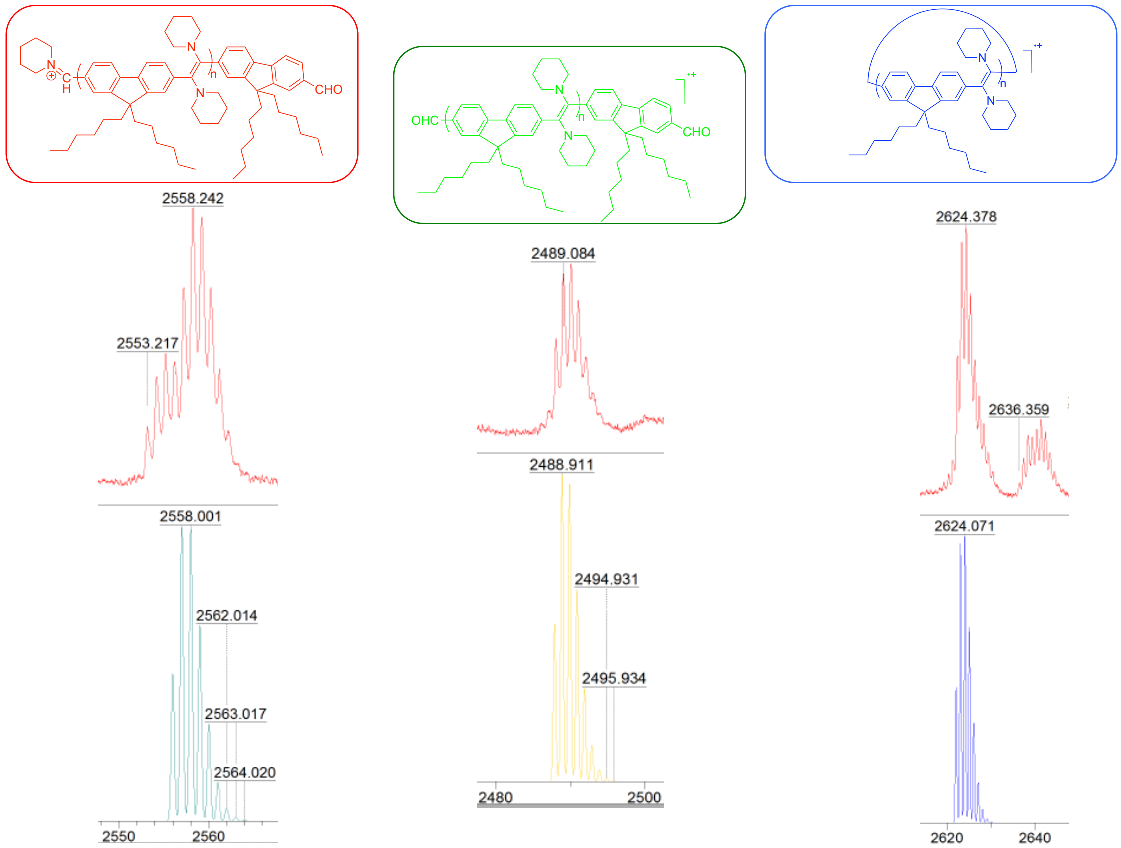

Supplementary Figure 20. MALDI spectra of compound **9c. a)** experimental; **b)** comparison of experimental (top) and theoretical isotopic profile between spectra (bottom)

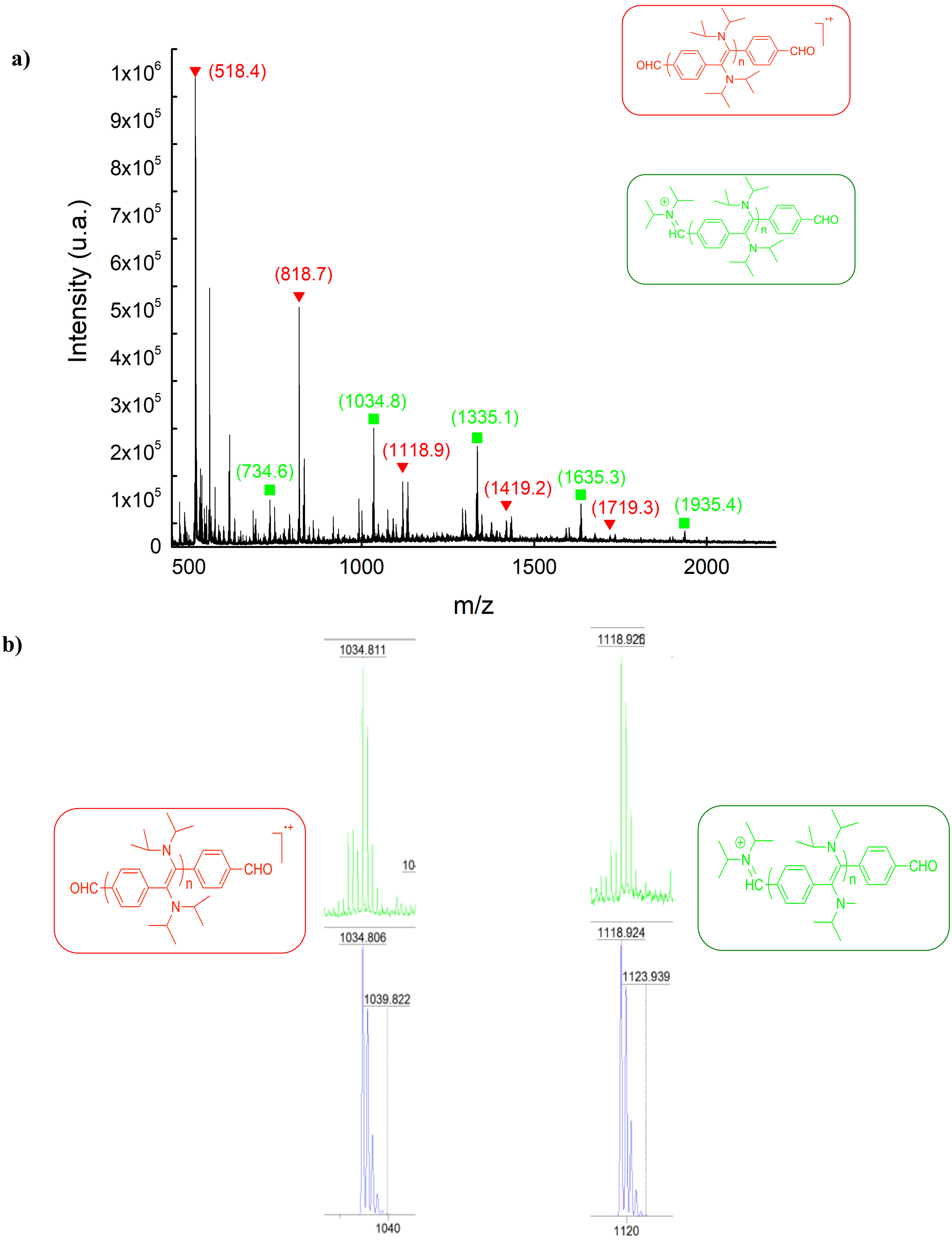

Supplementary Figure 21. MALDI spectrum of compound **9a. a)** experimental; **b)** comparison of experimental (top) and theoretical (bottom) isotopic profile.

2.9. Crystallographic data

Crystal data and structure refinement for **3a**:

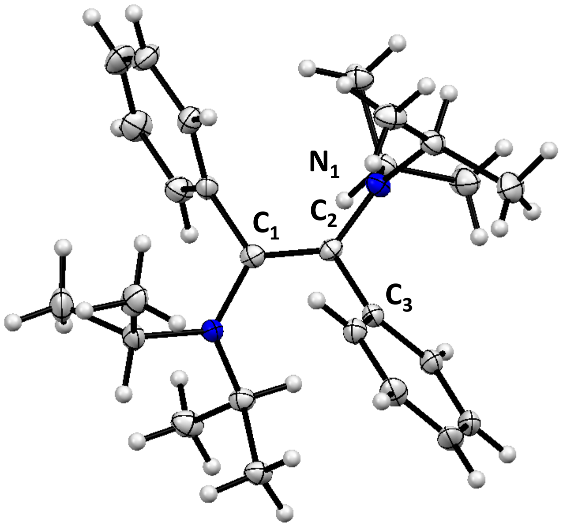

Identification code	1,2-bis(diisopropylamino)stilbene
Empirical formula	$C_{26}H_{38}N_2$
Formula weight	378.58
Temperature/K	373(2)
Crystal system	monoclinic
Space group	$P2_1/c$
a/Å	14.677(3)
b/Å	17.737(2)
c/Å	19.322(3)
α/°	90
β/°	112.105(4)
γ/°	90
Volume/Å^3	4660.0(13)
Z	8
ρ_{calc}g/cm^3	1.079
μ/mm^{-1}	0.465
F(000)	1664.0
Crystal size/mm^3	0.200 × 0.200 × 0.200
Radiation	CuKα (λ = 1.54178)
2Θ range for data collection/°	6.5 to 136.48
Index ranges	-17 ≤ h ≤ 17, -21 ≤ k ≤ 20, -23 ≤ l ≤ 23
Reflections collected	34019
Independent reflections	8527 [R_{int} = 0.0120, R_{sigma} = 0.0112]
Data/restraints/parameters	8527/0/521
Goodness-of-fit on F^2	1.252
Final R indexes [I>=2σ (I)]	R_1 = 0.0599, wR_2 = 0.1524
Final R indexes [all data]	R_1 = 0.0637, wR_2 = 0.1537
Largest diff. peak/hole / e Å^{-3}	0.27/-0.28

Crystal data and structure refinement for **3b**:

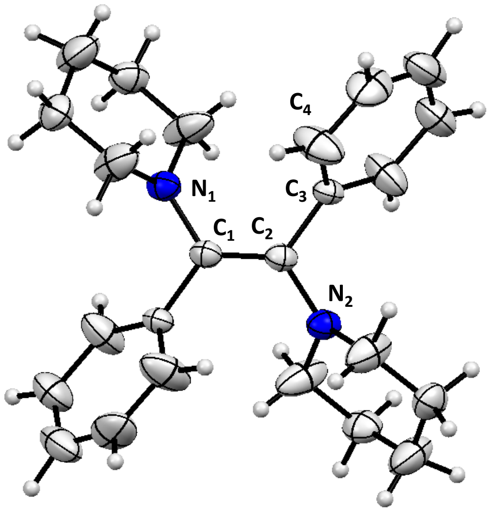

Identification code	1,2-bis(piperidine)stilbene
Empirical formula	C12H15N
Formula weight	173.25
Temperature/K	153(2)
Crystal system	monoclinic
Space group	C2/c
a/Å	18.199(8)
b/Å	5.945(3)
c/Å	18.968(13)
α/°	90
β/°	97.767(18)
γ/°	90
Volume/Å3	2033.4(19)
Z	8
ρcalcg/cm3	1.132
μ/mm-1	0.496
F(000)	752.0
Crystal size/mm3	0.060 × 0.040 × 0.010
Radiation	CuKα (λ = 1.54187)
2Θ range for data collection/°	15.696 to 136.384
Index ranges	-21 ≤ h ≤ 21, -7 ≤ k ≤ 7, -18 ≤ l ≤ 22
Reflections collected	5658
Independent reflections	1850 [Rint = 0.0319, Rsigma = 0.0337]
Data/restraints/parameters	1850/0/118
Goodness-of-fit on F2	1.097
Final R indexes [I>=2σ (I)]	R1 = 0.0771, wR2 = 0.1858
Final R indexes [all data]	R1 = 0.0963, wR2 = 0.1954
Largest diff. peak/hole / e Å-3	0.41/-0.41

Crystal data and structure refinement for **3c**:

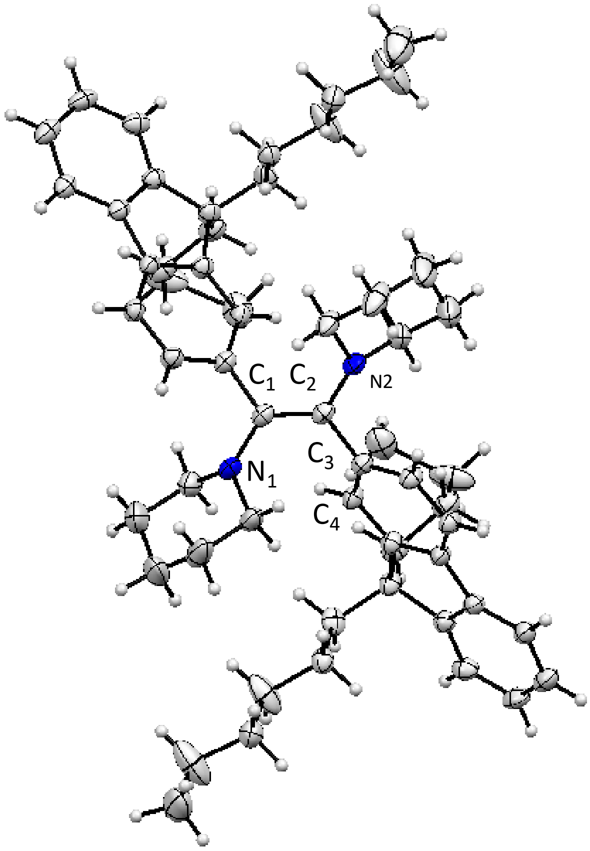

Identification code	1,2-bis(9,9-dihexyl-9H-fluorene)-1,2-bis(piperidine)ethene
Empirical formula	C64H90.5N2
Formula weight	887.88
Temperature/K	100(2)
Crystal system	monoclinic
Space group	C2/c
a/Å	26.645(3)
b/Å	10.2444(12)
c/Å	22.697(3)
α/°	90
β/°	114.360(3)
γ/°	90
Volume/Å3	5643.9(12)
Z	4
ρcalcg/cm3	1.045
μ/mm-1	0.437
F(000)	1954.0
Crystal size/mm3	0.200 × 0.200 × 0.200
Radiation	CuKα (λ = 1.54178)
2Θ range for data collection/°	7.284 to 145.562
Index ranges	-32 ≤ h ≤ 32, -12 ≤ k ≤ 9, -28 ≤ l ≤ 25
Reflections collected	21425
Independent reflections	5547 [Rint = 0.0196, Rsigma = 0.0194]
Data/restraints/parameters	5547/7/341
Goodness-of-fit on F2	1.103
Final R indexes [I>=2σ (I)]	R1 = 0.0598, wR2 = 0.1821
Final R indexes [all data]	R1 = 0.0711, wR2 = 0.1901
Largest diff. peak/hole / e Å-3	0.58/-0.48

Crystal data and structure refinement for **7a**:

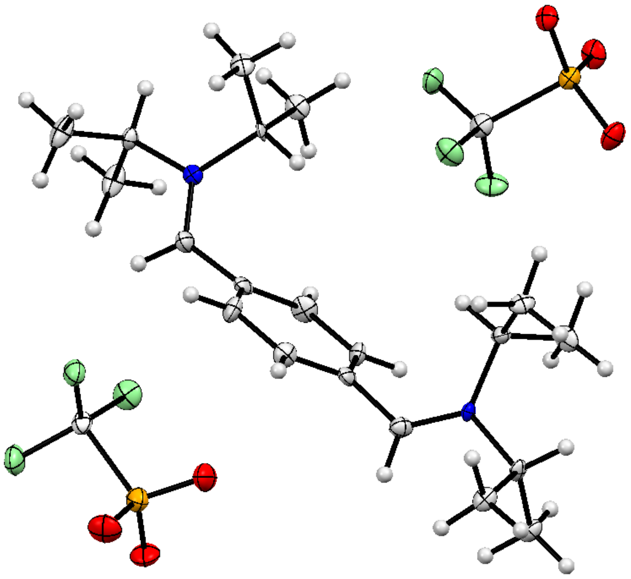

Identification code	Benzylidenebis(diisopropyliminium) triflate
Empirical formula	C22H34F6N2O6S2
Formula weight	600.63
Temperature/K	566.3
Crystal system	orthorhombic
Space group	Pna21
a/Å	21.464(16)
b/Å	12.915(11)
c/Å	9.932(8)
α/°	90
β/°	90
γ/°	90
Volume/Å3	2753(4)
Z	4
ρcalcg/cm3	1.449
μ/mm-1	2.484
F(000)	1256.0
Crystal size/mm3	0.2 × 0.2 × 0.2
Radiation	CuKα (λ = 1.54178)
2Θ range for data collection/°	7.99 to 136.448
Index ranges	-25 ≤ h ≤ 21, -15 ≤ k ≤ 12, -11 ≤ l ≤ 11
Reflections collected	13682
Independent reflections	4304 [Rint = 0.0863, Rsigma = 0.0605]
Data/restraints/parameters	4304/1/352
Goodness-of-fit on F2	1.055
Final R indexes [I>=2σ (I)]	R1 = 0.0605, wR2 = 0.1548
Final R indexes [all data]	R1 = 0.0617, wR2 = 0.1561
Largest diff. peak/hole / e Å-3	0.60/-0.45
Flack parameter	0.50(3)

Crystal data and structure refinement for **4a**:

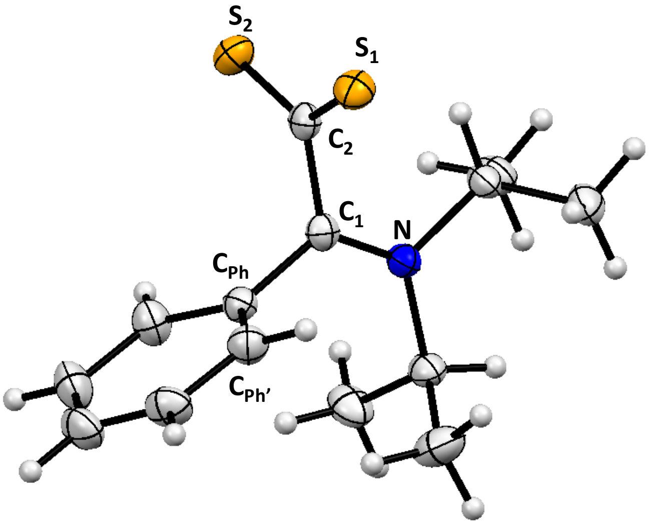

Identification code	Benzylidenediisopropylimnium-2-dithiocarboxylate
Empirical formula	C14H19NS2
Formula weight	265.42
Temperature/K	120(2)
Crystal system	triclinic
Space group	P-1
a/Å	7.5701(3)
b/Å	8.9559(3)
c/Å	11.8979(4)
α/°	106.304(3)
β/°	108.363(3)
γ/°	94.556(3)
Volume/Å3	722.20(5)
Z	2
ρcalcg/cm3	1.221
μ/mm-1	3.100
F(000)	284.0
Crystal size/mm3	0.1 × 0.01 × 0.01
Radiation	CuKα (λ = 1.54178)
2Θ range for data collection/°	8.274 to 136.48
Index ranges	-7 ≤ h ≤ 9, -10 ≤ k ≤ 10, -14 ≤ l ≤ 14
Reflections collected	10131
Independent reflections	2628 [Rint = 0.0217, Rsigma = 0.0142]
Data/restraints/parameters	2628/0/158
Goodness-of-fit on F2	1.092
Final R indexes [I>=2σ (I)]	R1 = 0.0281, wR2 = 0.0769
Final R indexes [all data]	R1 = 0.0290, wR2 = 0.0776
Largest diff. peak/hole / e Å-3	0.28/-0.19

Crystal data and structure refinement for **E-3b-(HOTf)₂**:

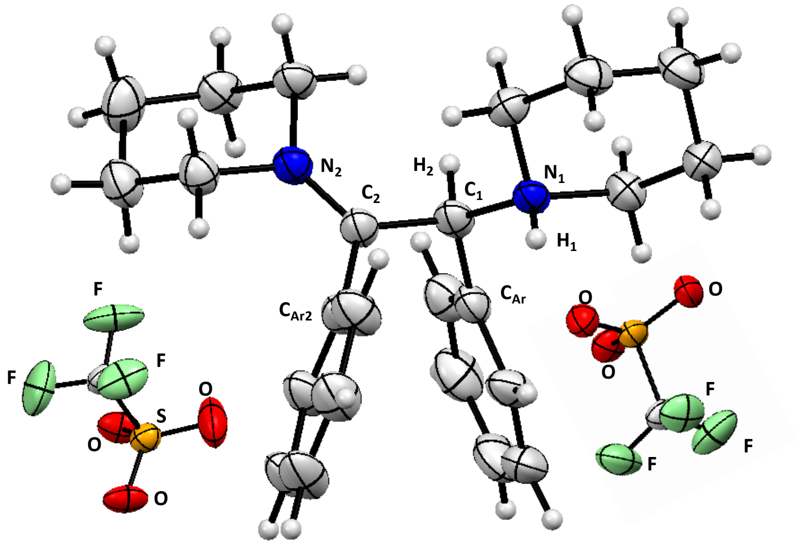

Identification code	1-piperidinium-2-piperidineiminium-1,2-(diphenyl)ethane bis triflate
Empirical formula	$C_{11}H_{10}FNOS$
Formula weight	172.20
Temperature/K	293(2)
Crystal system	triclinic
Space group	P-1
a/Å	9.8105(4)
b/Å	11.7978(6)
c/Å	13.4042(7)
α/°	79.358(5)
β/°	74.454(5)
γ/°	76.046(4)
Volume/Å^3	1438.56(13)
Z	6
ρ_{calc}g/cm^3	1.193
μ/mm^{-1}	0.614
F(000)	546.0
Crystal size/mm^3	? × ? × ?
Radiation	CuKα (λ = 1.54184)
2Θ range for data collection/°	13.048 to 136.494
Index ranges	-11 ≤ h ≤ 10, -14 ≤ k ≤ 14, -16 ≤ l ≤ 16
Reflections collected	15485
Independent reflections	5200 [R_{int} = 0.2484, R_{sigma} = 0.1136]
Data/restraints/parameters	5200/0/382
Goodness-of-fit on F^2	1.723
Final R indexes [I>=2σ (I)]	R_1 = 0.1616, wR_2 = 0.4074
Final R indexes [all data]	R_1 = 0.1669, wR_2 = 0.4236
Largest diff. peak/hole / e Å^{-3}	1.61/-0.68

Crystal data and structure refinement for **E-3a-(HOTf)$_2$**:

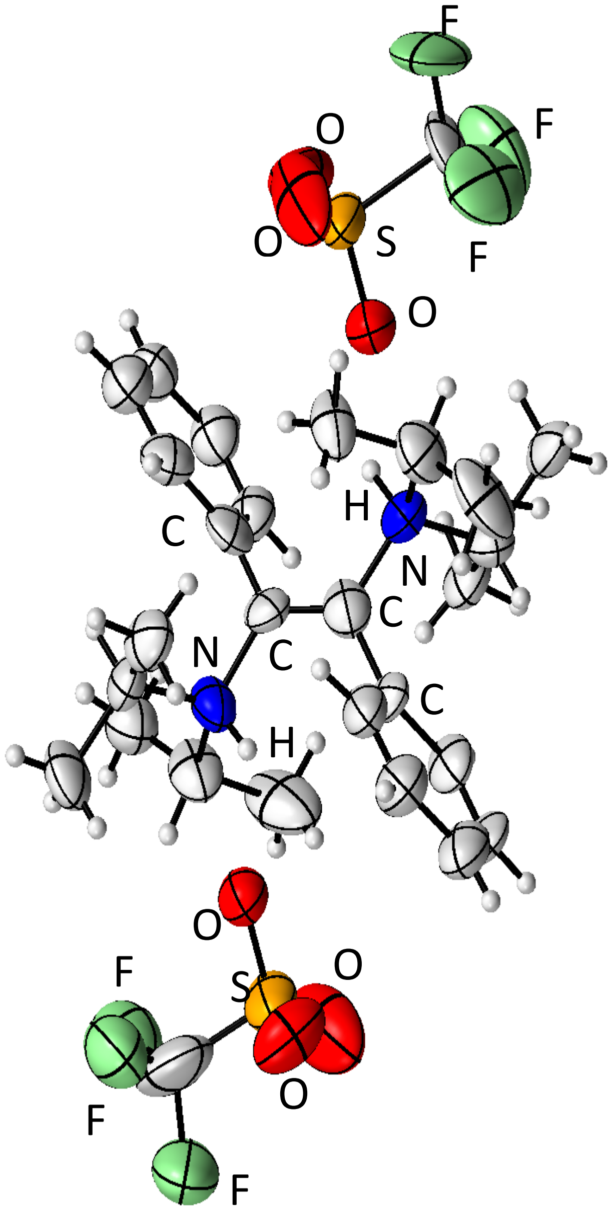

Identification code	(E)-1,2-bis(diisopropylammonium)stillbene bis triflate
Empirical formula	$C_{28}H_{40}F_6N_2O_6S_2$
Formula weight	678.74
Temperature/K	566(2)
Crystal system	monoclinic
Space group	Cc
a/Å	23.542(11)
b/Å	9.569(5)
c/Å	16.752(8)
α/°	90
β/°	98.967(9)
γ/°	90
Volume/Å^3	3728(3)
Z	4
ρ_{calc}g/cm^3	1.209

μ/mm^{-1}	1.849
F(000)	1424.0
Crystal size/mm^3	0.200 × 0.200 × 0.200
Radiation	CuKα (λ = 1.54178)
2Θ range for data collection/°	5.34 to 140.13
Index ranges	-28 ≤ h ≤ 28, -11 ≤ k ≤ 11, -20 ≤ l ≤ 20
Reflections collected	14119
Independent reflections	6105 [R_{int} = 0.0951, R_{sigma} = 0.1217]
Data/restraints/parameters	6105/20/406
Goodness-of-fit on F^2	0.820
Final R indexes [I>=2σ (I)]	R_1 = 0.0883, wR_2 = 0.2309
Final R indexes [all data]	R_1 = 0.1519, wR_2 = 0.2549
Largest diff. peak/hole / e Å^{-3}	0.52/-0.40
Flack parameter	0.18(8)

2.10 Computational details

All calculations were performed using the Gaussian 09 package[1-3] and the B3LYP hybrid[4] functional on the real systems. The def2-SVP[5] basis set was employed for all atoms. All stationary points involved were fully optimized. Frequency calculations were undertaken to confirm the nature of the stationary points, yielding one imaginary frequency for transition states (TS), corresponding to the expected process, and all of them positive for minima. The connectivity of the transition states and their adjacent minima was confirmed by intrinsic reaction coordinate (IRC)[6,7] calculations. All the geometrical structures were plotted with Gaussview 5.0.[8]

TD-DFT calculations have been performed at CAM-B3LYP/6-311++G** level of theory on the geometry optimized at B3LYP/def2-SVP in order to obtain the 1st transition energy (λ_{max}) for dimers **3a-c**, corresponding to HOMO→LUMO transition.

2.10.1 Calculations of HOMO-LUMO gap

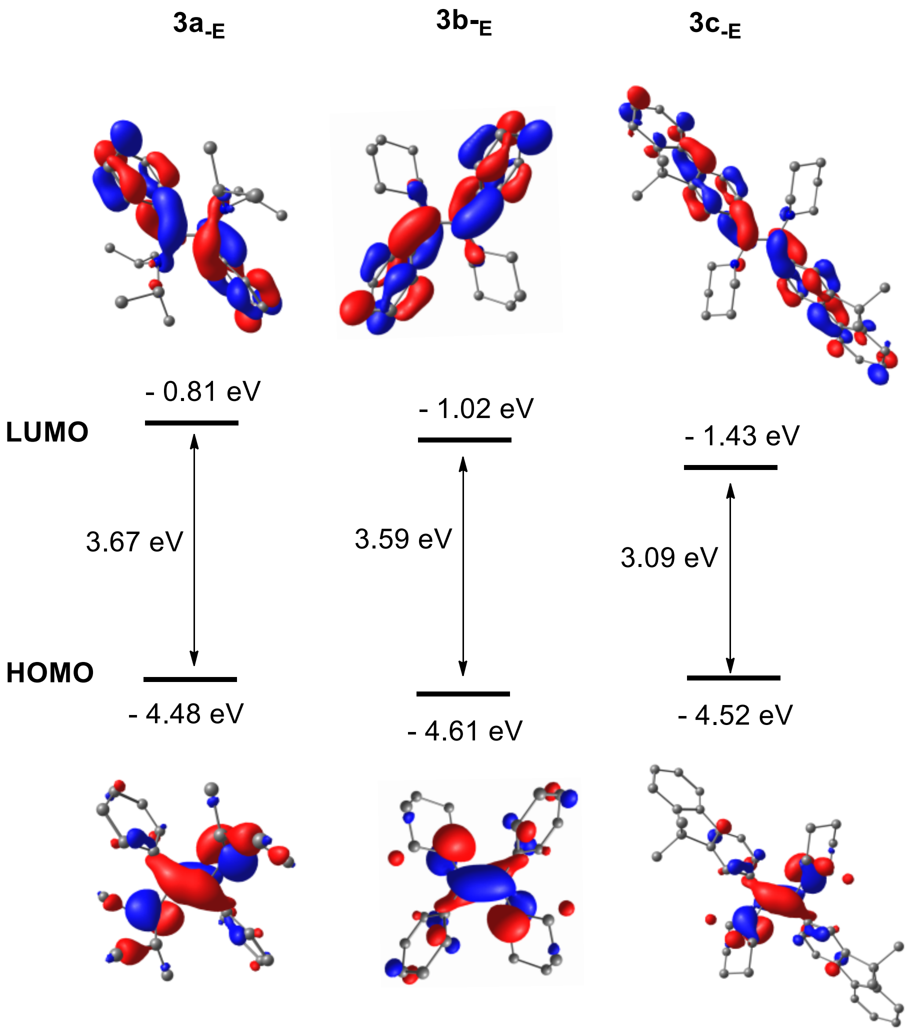

Supplementary Figure 22. Frontier orbitals for the E isomer of the 3 dimers **3a-c** (cutoff : 0.04). In the case of dimer **3c** the C_6H_{13} alkyl groups are replaced by CH_3 groups.

In each isomer, the HOMO is mainly localized on the $\pi_{C=C}$ and n_{N^-} orbitals while the LUMO is localized on the combination of the orbitals π^*_{C-N} and $\pi^*_{C=C}$ on the phenyl ring.
Moreover, the values obtained for the HOMO-LUMO gap have the same tendency found experimentally, with the highest gap found for **3a**>**3b**>**3c**. These results are in agreement with the maximum of absorption observed with a λ_{max} of **3c**>**3b**>**3a**.

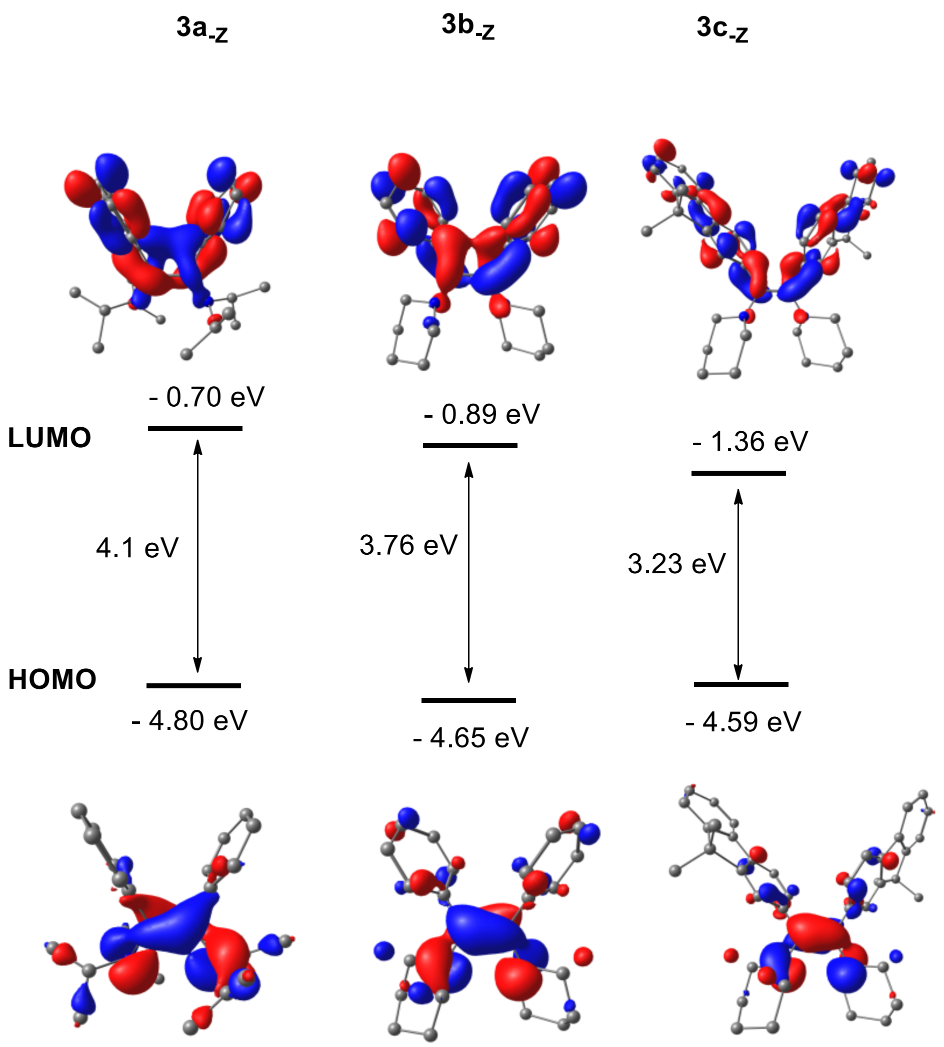

Supplementary Figure 23. Frontier orbitals for the Z isomer of the 3 dimers **3a-c** (cutoff: 0.04).). In the case of dimer **3c** the C_6H_{13} alkyl groups are replaced by CH_3 groups.

In each isomers, the HOMO is mainly localized on the $\pi_{C=C}$ and n_{N^-} orbitals while the LUMO is localized on the combination of the orbitals π^*_{C-N} and $\pi^*_{C=C}$ on the phenyl ring.

dimer	HOMO →LUMO transition	Gap in eV
3a_E	356.46 (f : 0.1684)	3.48
3a_Z	315.29 (f : 0.0988)	3.93
3b_E	366.02 (f : 0.2320)	3.39
3b_Z	340.98 (f : 0.2574)	3.63
3c_E	398.25 (f : 0.6165)	3.11
3c_Z	376.35 (f : 0.5024)	3.29

Supplementary Table 2. TD-DFT calculations. HOMO → LUMO excitation energy (λ_{max} in nm) and oscillator strengths (f) calculated at CAM-B3LYP/6-311++G** level of theory on the geometry optimized at B3LYP/def2-SVP. Associated gap in eV.

The trend in the maximum of absorption found by calculations is in agreement with the one obtained experimentally. Indeed, the maximum of absorption increases from **3a** to **3c** compounds.

2.10.2 Torsion angles and main bond lengths

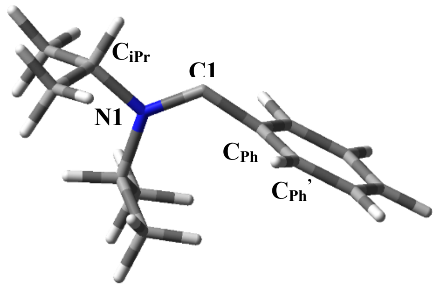

2a-Singlet

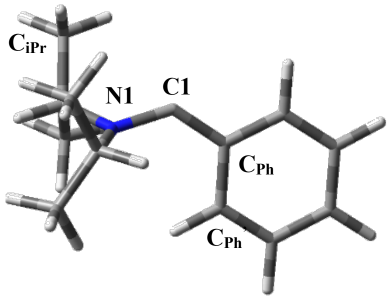

2a-Triplet

	2a-Singlet	2a-Triplet
**ΔG_{S-T} (ΔE_{S-T})**	0 (0)	18.5 (19.8)
**C1-N1**	1.304	1.367
**C1-CPh**	1.457	1.414
**N1-C1-CPh**	124.23	129.78
**ΣN1**	360.0	355.7
**CiPr-N1-C1-CPh**	180.0	-110.2
**CPh'-CPh-C1-N1**	95.1	-178.4

Supplementary Table 3. Relative stability of singlet (S) and triplet (T) states for carbene **2a** computed at B3LYP/def2-SVP level of theory. Gibbs free energy and electronic energy into bracket, in kcal.mol^{-1}. Main geometrical parameters, bond lengths in Å and main bond angles in °.

As expected, the singlet state is more stable than the triplet state around 18.5 kcal.mol^{-1}.

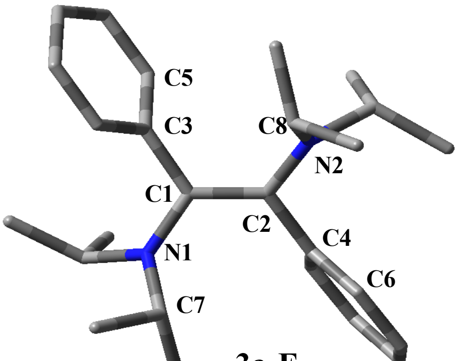

3a-E

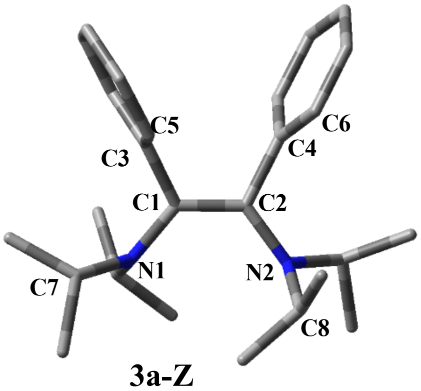

3a-Z

	3a-E	3a-Z
ΔG (ΔE)	0 (0)	9.7 (10.5)
**C1-C2**	1.377	1.380
**C1-N1**	1.421	1.427
**C2-N2**	1.420	1.427
**C1-C3**	1.504	1.519
**C2-C4**	1.504	1.519
**N1-C1-C2**	122.08	126.90
**N1-C1-C3**	116.72	116.60
**C3-C1-C2**	121.01	116.40
**N2-C2-C1**	122.09	126.90
**N2-C2-C4**	116.73	116.60
**C4-C2-C1**	121.01	116.40
**ΣN1**	359.8	353.6
**ΣN2**	359.8	353.6
**C5-C3-C1-C2**	66.8	-76.8
**C6-C4-C2-C1**	-115.9	104.1
**C7-N1-C1-C2**	53.0	135.4
**C8-N2-C2-C1**	53.0	-74.0

Supplementary Table 4. Relative stability of Z and E forms for dimer **3a** computed at B3LYP/def2-SVP level of theory. Gibbs free energy and electronic energy into bracket, in kcal.mol^{-1}. Main geometrical parameters, bond lengths in Å and main bond angles in °. Hydrogen atoms have been omitted for clarity.

The parameters calculated for the dimer **3a-E** are in agreement with the ones observed by X-Ray analysis.

2.10.3 Direct dimerization

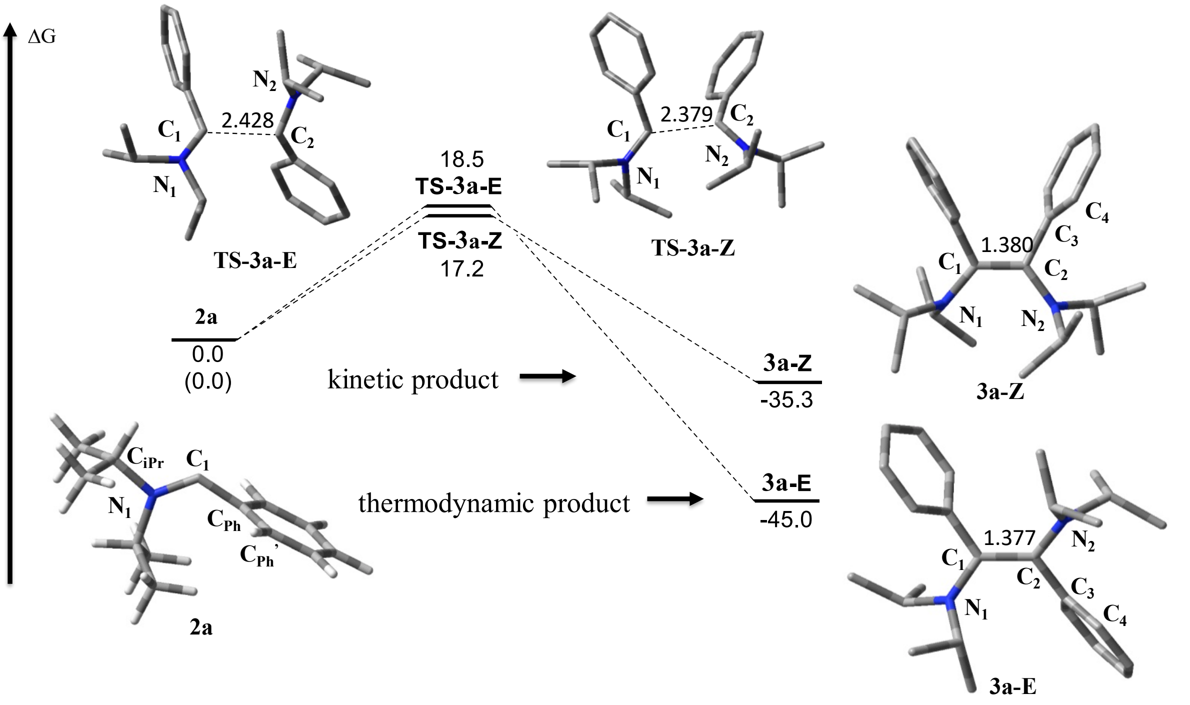

Supplementary Figure 24. Energy profiles computed for the dimerization of **2a**, using B3LYP/def2-SVP level theory. Gibbs free energy in kcal.mol^{-1}.

The direct dimerization of the carbene into the alkene, with both the Z- and the E-configuration, was found strongly exergonic, with ΔG of -35 kcal.mol^{-1} for **3a-Z** and -45 kcal.mol^{-1} for **3a-E**. **3a-E** is

thermodynamically more stable by 10 kcal.mol^{-1} than **3a-Z**, which could explain the E/Z ratio in favor of the E-isomer observed experimentally (thermodynamic control).

Moreover, the potential energy surface of the transformation **2a** into **3a-E/Z** was scrutinized. Two transition states connecting **2a** and **3a-E**, namely **TS-3a-E**, and **2a** and **3a-Z**, namely **TS-3a-Z**, could be localized, leading to close and moderate activation barriers of 18.5 and 17.2 kcal.mol^{-1} respectively.

For **TS-3a-E**, the $C_1{\cdots}C_2$ bond (2.428 Å) is strongly elongated compared to its corresponding dimer **3a-E** (76 %). Moreover, the C_1-N_1 bond of 1.31 Å, the planar nitrogen and carbon atoms (ΣN_1 = 360° and carbon ΣC_1 = 360°, respectively) found in the carbene on the left part of TS-3a-E are close to the geometrical parameters of the starting carbene **2a**. Similar geometrical parameters are observed for the carbene on the right part of **TS-3a-E**, with a C_2-N_2 bond distance of 1.33 Å, a ΣN_1 of 359.9° and a ΣC_1 of ~ 359.0°. Thus, **TS-3a-E** can be considered as an early transition state.

For **TS-3a-Z**, the $C_1{\cdots}C_2$ bond (2.379 Å) is strongly elongated compared to its corresponding dimer **3a-Z** (72 %) but less than the one in **TS-3a-E**, suggesting a slightly stronger interaction between the two carbenes in **TS-3a-Z**. As a result, $C_{1,2}$ and $N_{1,2}$ are moderately pyramidalized (353° and 359.7° for $\Sigma C_{1,2}$ and $\Sigma N_{1,2}$ respectively) and $C_{1,2}$-$N_{1,2}$ bonds are slightly longer (1.33 Å for each C-N bond). Although those data are in agreement with an early transition state, **TS-3a-Z** is relatively later than **TS-3a-E**.

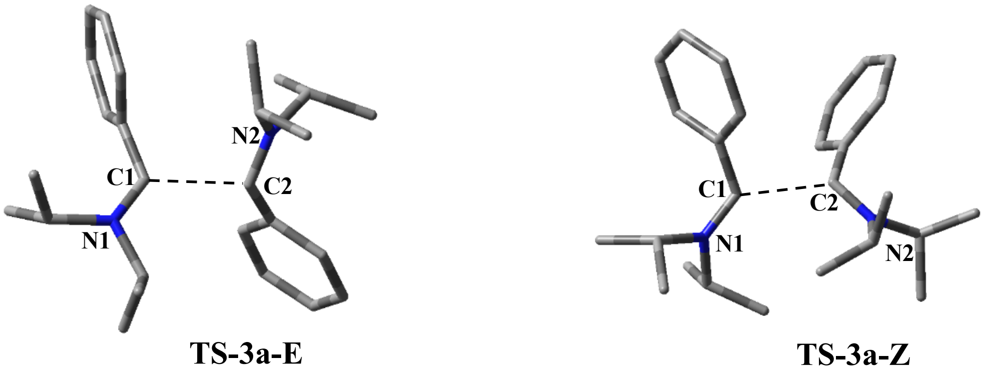

	TS-3a-E	TS-3a-Z
**C1-C2**	2.428	2.379
**C1-N1**	1.306	1.327
**C2-N2**	1.326	1.327
**C1-C3**	1.457	1.470
**C2-C4**	1.461	1.470
**C3-C1-N1**	124.22	117.37
**N2-C2-C4**	123.21	117.37
**N1-C1-C2-N2**	-133.6	7.8
**ΣN1**	360.0	359.7
**ΣN2**	359.9	359.7

Supplementary Table 5. Main geometrical parameters (bond lengths in Å and main bond angles in °) for the two transition states (**TS-3a-Z** and **TS-3a-E**) associated to dimerization process from carbene **2a**. Hydrogen atoms have been omitted for clarity.

2.10.4 Side reaction (intramolecular C-H insertion)

Intramolecular C-H insertion reactions of aminoarylcarbene **2a** into CHiPr or CH_3iPr were predicted to be exergonic (ΔG = -16 and -30.6 kcal.mol^{-1} respectively). Prohibitive activation barriers of 39.4 kcal.mol^{-1} and 45.6 kcal.mol^{-1} respectively, were calculated, in agreement with the high selectivity observed experimentally for the dimerization reaction.

a) C-H Insertion in β position

Insertion of carbene **2a** into the CH_3iPr was first investigated. A single transition state connecting **2a** and the cyclobutane **6a**, namely **TS-6a**, could be localized on the potential energy surface. Analysis of the geometrical parameters of **TS-6a** revealed that the disruptive H_3-CiPr' bond was elongated by only 18 % compared to that of **2a** (1.29 Å vs 1.09 Å) and that the forming C_{2a}-H bond was also elongated by 18 % compared to the C_{2a}-H bond of **6a** (1.32 Å vs 1.11 Å). In parallel, the forming C_{2a}-CiPr' bond was moderately elongated by 37 % compared to that of cyclobutane **6a** (2.12 Å vs 1.54 Å). As a consequence of the C_{2a}-CiPr' interaction, both C_{2a} and N_{2a} atoms are strongly pyramidalized (ΣN_{2a} = 332° and ΣC_{2a} = 333°, respectively), and the C_{2a}-N_{2a} bond length of 1.43 Å is characteristic of a C-N single bond (1.42 Å). Therefore, **TS-6a** can thus be considered as a late transition state. Overall, these data suggest that this insertion reaction is a concerted but asynchronous process.

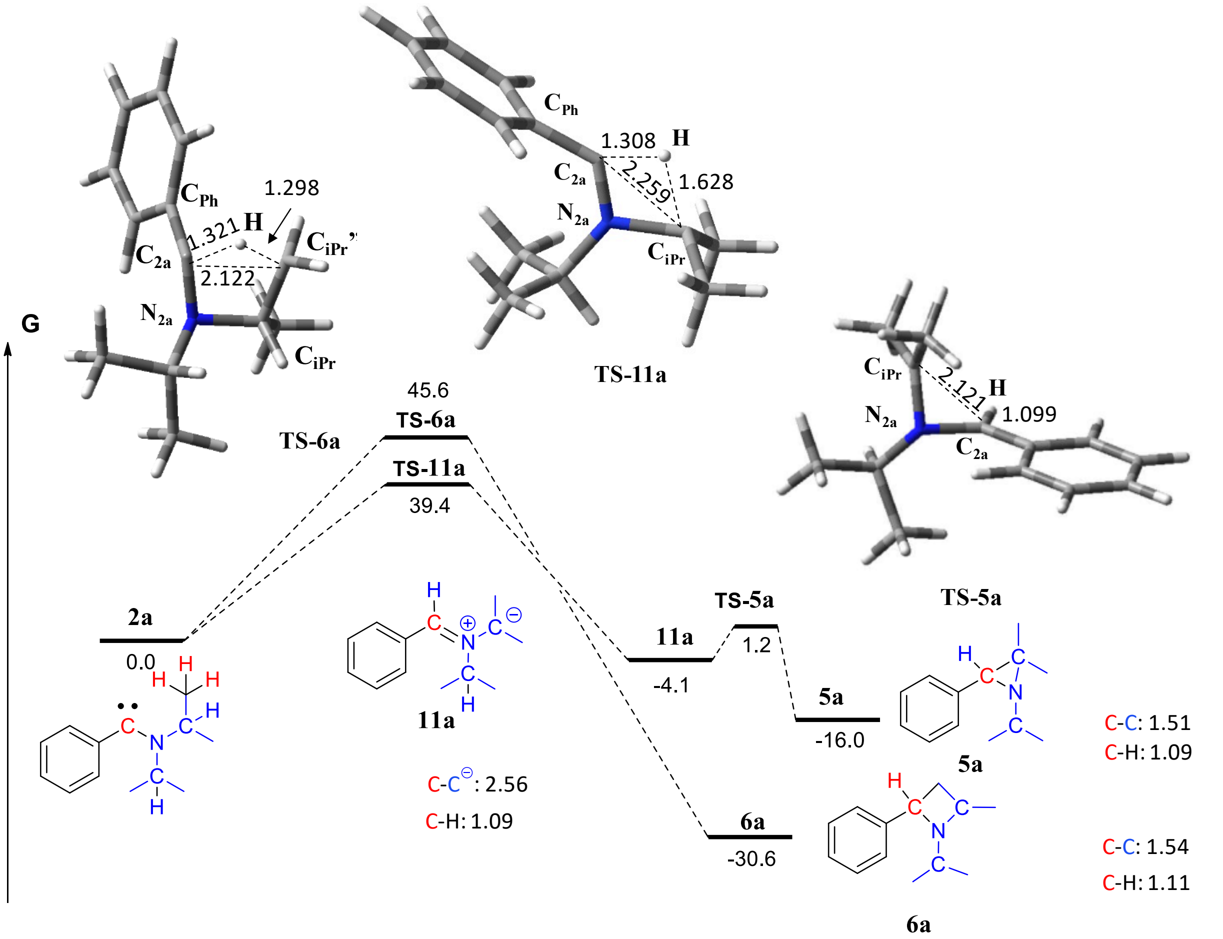

Supplementary Figure 25. Energy profiles computed for the C-H insertion reactions of **2a** at B3LYP/def2-SVP level of theory. Gibbs free energy in kcal.mol^{-1} and bond distance in Å.

b) C-H insertion in α position

The insertion of carbene **2a** into CHiPr was next investigated. Insertion of carbene **2a** into CHiPr involves a stepwise process, where the CHiPr proton of **2a** is transferred to the carbene center (C_{2a}) with a high activation barrier of 39.4 kcal.mol^{-1} (**TS-11a**) for the first step (**2a→11a**); the resulting azomethine ylide intermediate **11a** then undergoes a ring closure in the second step (**11a→5a**) with a very low activation barrier of 5.3 kcal.mol^{-1} (**TS-5a)**.

Analysis of **TS-11a** allows some insight into the nature of this proton transfer step to be gained. The C-HiPr bond in **TS-11a** is strongly elongated by 48 % compared to that of the starting carbene **2a** (1.62 Å vs 1.09 Å), while the forming C_{2a}-H bond is only moderately elongated (21 %), compared to the C_{2a}-H of compound **11a** (1.30 Å vs 1.09 Å). In parallel, the C_{2a}-C_{iPr} bond distance remains long (2.26 Å) in comparison with that of the azomethine ylide **11a** (2.56 Å). Besides, the short C_{2a}-N_{2a} bond distance of 1.30 Å, the sum of angles around C_{2a} and N_{2a} of 360° attest of the absence of any re-hybridization of those atoms between **2a** and **11a**. From those data, it can be concluded that **TS-11a** is a late transition state.

The cyclopropane final compound is formed in the last step of this process by ring closure of the zwitterionic azomethine ylide **1a**. This step proceeds via **TS-5a**, where the forming C_{2a}-C_{iPr} is strongly elongated by 40 % compared to that of the final compound **5a** (2.12 Å vs 1.51 Å). The long C_{2a}-N_{2a} bond distance of 1.43 Å (characteristic of a single C-N bond) associated with the pyramidal N_{2a} atom (ΣN_{2a} = 339°) and the planar C_{2a} atom (ΣC_{2a} = 360°) suggest a zwitterionic structure for **TS-5a**, where both the negative and positive charges are localized at C_{iPr} and C_{2a}, respectively.

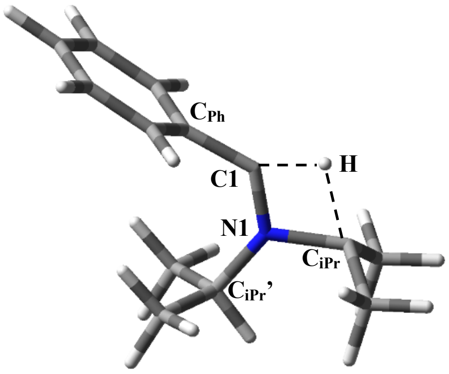

TS-5a

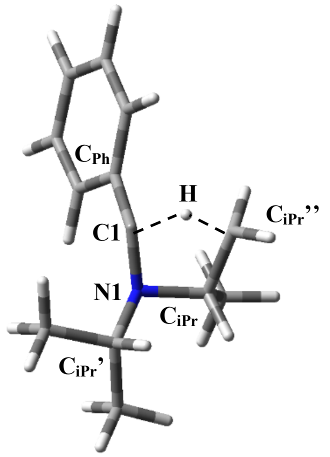

TS-11a

	**TS-5a**	**TS-11a**
**C1-N1**	1.295	1.429
**C1-H**	1.308	1.321
**C_{iPr}-H**	1.628	/
**C_{iPr}-N1**	1.527	1.478
**C1-C_{Ph}**	1.469	1.464
**C_{iPr}'-N1**	1.486	1.480
**C_{iPr}'-C_{iPr}''**	/	1.552
**C_{iPr}''- H**	/	1.298
**C1-N1-C_{iPr}**	106.04	102.14
**N1-C_{iPr}-H**	68.21	/
**C1-H-C_{iPr}**	100.03	/
**C1-N1-C_{iPr}'**	130.80	115.01
**N1-C1-H**	85.72	105.57
**C1-H-C_{iPr}''**	/	108.30
**H-C_{iPr}''-C_{iPr}'**	/	103.17

Supplementary Table 6. Main geometrical parameters (bond lengths in Å and main bond angles in °) for the two transition states associated to insertion reaction into hydrogens α or β, respectively **TS-5a** and **TS-11a**.

2.10.5 Side reaction (elimination of propene from 3a)

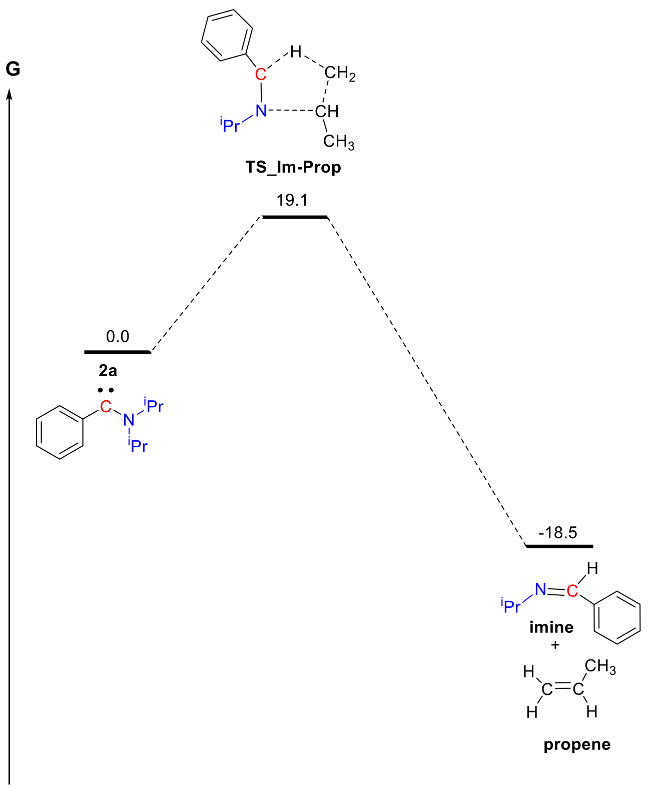

Supplementary Figure 26. Energy profile computed for the intra-molecular β-H elimination in **2a**, affording the corresponding imine and propene at B3LYP/def2-SVP level of theory. Gibbs free energy in $kcal.mol^{-1}$.

	TS_Im-Prop
**C1-N1**	2.012
**C1-C2**	1.427
**C2-H**	1.326
**C_{Ph}-H**	1.475
**C_{iPr}-N1**	1.255
**N1-C1-C2**	99.84
**C1-C2-H**	97.87
**C2-H-C_{Ph}**	139.13
**H-C_{Ph}-N1**	96.12

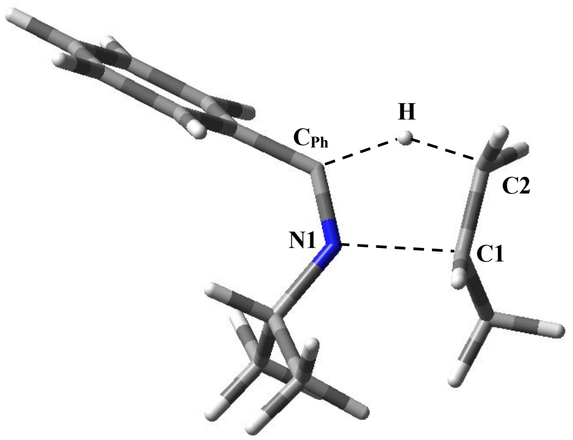

TS_Im-Prop

Supplementary Table 7. Main geometrical parameters (bond lengths in Å and main bond angles in °) for the transition state associated to β-H elimination, **TS_Im-Prop**.

Z-matrices and energy in au

2a-Singlet

Sum of electronic and zero-point Energies= -560.837748
Sum of electronic and thermal Free Energies = -560.879266

C	-1.20427300	0.00084000	-0.63759900
C	-1.92921500	-1.21002300	-0.51472100
C	-1.92945800	1.21129400	-0.51215400
C	-3.29494600	-1.20461500	-0.22719600
C	-3.29519100	1.20500200	-0.22465400
C	-3.99114800	-0.00003800	-0.07262500
H	-1.40958900	-2.16151200	-0.65831200
H	-1.41003000	2.16319000	-0.65374900
H	-3.82346200	-2.15778500	-0.12831200
H	-3.82390100	2.15785400	-0.12376400
H	-5.06181200	-0.00037800	0.14703700
C	0.17995800	0.00145900	-1.09269500
N	1.21351600	0.00061700	-0.29764400
C	2.56148600	0.00125000	-0.95789400
H	2.30104800	0.00241900	-2.02464800
C	3.34993400	-1.27585200	-0.65563200
C	3.35014200	1.27757400	-0.65288800
H	2.75888500	2.17244800	-0.90289000
H	3.65892500	1.34758000	0.40325400
H	4.26763000	1.29813300	-1.26253400
H	3.65868000	-1.34818400	0.40036400
H	4.26742900	-1.29524400	-1.26530600
H	2.75853400	-2.17008900	-0.90756900
C	1.25018000	-0.00137600	1.21369700
H	2.31928200	-0.00136500	1.47084200
C	0.64723200	-1.27709800	1.81002900
C	0.64634400	1.27233700	1.81342100
H	1.09671100	2.17529300	1.37277100
H	-0.44055300	1.31846100	1.66248900
H	0.84091300	1.29229100	2.89839700
H	-0.43963100	-1.32359400	1.65896900
H	0.84181400	-1.29980000	2.89494800
H	1.09824500	-2.17855800	1.36697900

2a-Triplet

Sum of electronic and zero-point Energies= -560.806265
Sum of electronic and thermal Free Energies= -560.849797

C	1.34694500	-0.09940200	-0.39928000
C	2.38848400	-0.48355000	-1.29904000
C	1.73989900	0.48558500	0.84672900
C	3.72520500	-0.29618100	-0.97136200
C	3.08222100	0.67129600	1.15824800
C	4.08670700	0.28327500	0.25712400
H	2.10756400	-0.93061500	-2.25623400
H	0.96125600	0.78392300	1.55296400
H	4.50315500	-0.60113700	-1.67766800
H	3.35689000	1.12188800	2.11708000
H	5.14030000	0.43064700	0.50887900
C	-0.00811200	-0.28978700	-0.75690400
N	-1.14716100	0.02768900	-0.07022800
C	-1.77800200	1.34071700	-0.35406600
H	-0.96201600	1.95037100	-0.77290800
C	-2.25464900	2.02718100	0.93234700
C	-2.88720400	1.26216700	-1.41348600
H	-2.51160700	0.79362100	-2.33614500
H	-3.75424000	0.68215600	-1.05622200
H	-3.24831700	2.27293000	-1.66482700
H	-3.08740300	1.48409200	1.40877500
H	-2.61558600	3.04369000	0.70610500
H	-1.43573300	2.10881800	1.66354700
C	-1.97649500	-1.06894700	0.48400900
H	-2.92083000	-0.60317200	0.80738100
C	-1.30510100	-1.68027100	1.72041500
C	-2.31251200	-2.14417400	-0.56102800
H	-2.81397000	-1.71011700	-1.43894200
H	-1.39546000	-2.64384500	-0.91058900
H	-2.97817400	-2.90722100	-0.12552600
H	-0.32985600	-2.12054700	1.45724500
H	-1.93429800	-2.47762900	2.14854100
H	-1.13586500	-0.91912400	2.49789600

3a.E

Sum of electronic and zero-point Energies= -1121.772185
Sum of electronic and thermal Free Energies= -1121.830221

C	-1.94459900	-0.00042600	-0.18779200
C	-2.39456100	0.57225100	-1.39096900
C	-2.88477900	-0.18339800	0.84318200
C	-3.72950400	0.95304600	-1.55861800
C	-4.21723900	0.20598400	0.68658300
C	-4.64727000	0.77475700	-0.51776200
H	-1.68257300	0.70833800	-2.20759400
H	-2.56388600	-0.63942200	1.78306200
H	-4.05542200	1.38785100	-2.50806700
H	-4.92572600	0.06178400	1.50752000
H	-5.69164700	1.07300300	-0.64519800
C	-0.51777400	-0.45342300	-0.04061300
N	-0.30861500	-1.83761000	0.20015500
C	0.41780800	-2.28895900	1.40437300
H	0.80999700	-1.37175600	1.86414200
C	1.62398900	-3.19670100	1.10536600
C	-0.49979600	-2.95861600	2.44662800
H	-1.33743000	-2.30237200	2.72951500
H	-0.92193400	-3.90544400	2.07138900
H	0.06879300	-3.19500700	3.36172400
H	1.31309300	-4.18499200	0.72710300
H	2.19775800	-3.37581900	2.02935600
H	2.29780700	-2.74135900	0.36738900
C	-0.90361300	-2.87910500	-0.66055500
H	-0.42218100	-3.81391200	-0.33534700
C	-0.53084400	-2.69505800	-2.13864800
C	-2.42202900	-3.12098000	-0.51475200
H	-2.72442100	-3.18174000	0.54050200
H	-3.02085100	-2.33383900	-0.99372000
H	-2.68420000	-4.07801700	-0.99757300
H	-0.92277800	-1.74480600	-2.53465400
H	-0.95674700	-3.50964500	-2.74718800
H	0.56033900	-2.69990600	-2.27467400
C	1.94464100	0.00042300	-0.18772100
C	2.39464100	-0.57225900	-1.39088100
C	2.88479400	0.18341400	0.84327600
C	3.72959400	-0.95303100	-1.55849800
C	4.21726500	-0.20594500	0.68670900
C	4.64733400	-0.77471700	-0.51762400
H	1.68267300	-0.70836500	-2.20752300
H	2.56387400	0.63944100	1.78314500
H	4.05554200	-1.38783600	-2.50793700
H	4.92573200	-0.06172700	1.50766100
H	5.69171900	-1.07294700	-0.64503400
C	0.51780700	0.45340700	-0.04058600
N	0.30861900	1.83759200	0.20017300
C	-0.41794300	2.28892400	1.40431400
H	-0.81015000	1.37171100	1.86404800
C	-1.62412500	3.19661800	1.10516500
C	0.49952400	2.95862600	2.44666000
H	1.33714800	2.30241300	2.72964700
H	0.92167300	3.90546200	2.07145500
H	-0.06917200	3.19501100	3.36169000
H	-1.31322500	4.18491200	0.72691200
H	-2.19799500	3.37573600	2.02909200
H	-2.29785100	2.74123700	0.36712800
C	0.90366600	2.87910400	-0.66048500
H	0.42218300	3.81389700	-0.33531100
C	0.53102600	2.69506100	-2.13861000

C	2.42206300	3.12102100	-0.51455000
H	2.72436300	3.18178800	0.54072900
H	3.02094800	2.33389800	-0.99347000
H	2.68424900	4.07806600	-0.99734700
H	0.92300600	1.74481600	-2.53458800
H	0.95696900	3.50965700	-2.74710900
H	-0.56014600	2.69989500	-2.27472900

3a.Z
Sum of electronic and zero-point Energies= -1121.755508
Sum of electronic and thermal Free Energies= -1121.814738

C	-1.37921900	1.17800100	0.18966400
C	-1.91976900	1.95947000	-0.84771000
C	-1.51011000	1.66224600	1.50403500
C	-2.56294700	3.17135700	-0.58178200
C	-2.16504900	2.86751500	1.77851300
C	-2.69572800	3.62856400	0.73387300
H	-1.82022100	1.62699900	-1.88120100
H	-1.08564400	1.08084200	2.32737100
H	-2.96035800	3.76521100	-1.41007300
H	-2.25482100	3.21387900	2.81220900
H	-3.20382700	4.57450400	0.94123900
C	-0.68890800	-0.15551000	-0.04077900
N	-1.53529300	-1.28632000	-0.24528400
C	-2.75216400	-1.41545300	0.59227800
H	-2.51889500	-0.88352200	1.52725000
C	-3.04550800	-2.87890600	0.97444900
C	-4.03376900	-0.78285700	0.01131900
H	-3.90201400	0.28155800	-0.22397000
H	-4.35641000	-1.30367400	-0.90543700
H	-4.85845400	-0.86925600	0.73821600
H	-3.39409500	-3.46743100	0.10993800
H	-3.84946300	-2.91406100	1.72787300
H	-2.16241000	-3.38068100	1.38989500
C	-1.61800200	-1.91828500	-1.58398600
H	-2.50929100	-2.56122800	-1.53229300
C	-0.44417400	-2.84280200	-1.91708500
C	-1.85535900	-0.94214400	-2.74991500
H	-2.75736100	-0.33246600	-2.59892700
H	-0.99592700	-0.26537700	-2.88077900
H	-1.97942100	-1.50180500	-3.69175300
H	0.47638400	-2.26740100	-2.08054100
H	-0.65906300	-3.40630300	-2.84086100
H	-0.25839000	-3.56171900	-1.10704400
C	1.37933400	1.17784200	-0.18970400
C	1.91988200	1.95928800	0.84768800
C	1.51039900	1.66202100	-1.50408200
C	2.56321000	3.17109800	0.58177400
C	2.16549300	2.86720800	-1.77854600
C	2.69615600	3.62824200	-0.73388600
H	1.82020600	1.62686400	1.88118300
H	1.08595300	1.08062500	-2.32743500
H	2.96061000	3.76494000	1.41007800
H	2.25540000	3.21352200	-2.81224700
H	3.20437500	4.57412000	-0.94124200
C	0.68886900	-0.15558800	0.04073500
N	1.53513200	-1.28647700	0.24528100
C	2.75199500	-1.41575000	-0.59226700
H	2.51877700	-0.88384400	-1.52726700
C	3.04522500	-2.87924600	-0.97435800
C	4.03363900	-0.78322300	-0.01132000
H	3.90196000	0.28121800	0.22389600
H	4.35621700	-1.30400600	0.90547800
H	4.85833200	-0.86973400	-0.73819300
H	3.39373100	-3.46776000	-0.10980600
H	3.84920100	-2.91450800	-1.72775400
H	2.16209600	-3.38096400	-1.38980700
C	1.61778300	-1.91840900	1.58400200
H	2.50901000	-2.56143700	1.53232500
C	0.44387100	-2.84280400	1.91714700
C	1.85524800	-0.94224800	2.74989200
H	2.75734300	-0.33270800	2.59889600
H	0.99591100	-0.26535000	2.88070000
H	1.97920800	-1.50188100	3.69176000
H	-0.47662600	-2.26730900	2.08060600
H	0.65872400	-3.40630100	2.84093500
H	0.25800100	-3.56172400	1.10712900

TS-3a-E
Sum of electronic and zero-point Energies= -1121.667740
Sum of electronic and thermal Free Energies= -1121.729054

C	2.00290000	-0.78809900	-0.37906100
C	1.53365600	-1.27898900	-1.62182100
C	3.26867100	-1.27372800	0.05798900
C	2.27548500	-2.20261900	-2.36921100
C	4.00726200	-2.17862700	-0.69834900
C	3.51596600	-2.66054100	-1.92215800
H	0.58394300	-0.90798200	-2.00543100
H	3.65382600	-0.94397800	1.02724900
H	1.87664100	-2.55407200	-3.32629800
H	4.97563600	-2.52448700	-0.32317000
H	4.09458000	-3.37618300	-2.51220700
C	1.24063300	0.00942800	0.57903500
N	1.44852500	1.30394100	0.77417100
C	0.96902800	1.84334200	2.08943300
H	0.12155200	1.18782300	2.33644400
C	0.46388300	3.29078000	2.06284000
C	2.03483200	1.64907700	3.17459200
H	2.34652800	0.59412700	3.21543200
H	2.92524600	2.27151000	2.98441800
H	1.63676200	1.93182400	4.16344400
H	1.28282900	4.02789600	2.04030300
H	-0.11048400	3.48279100	2.98387600
H	-0.19909800	3.48178400	1.20642100
C	2.17757600	2.25879500	-0.13071400
H	1.99811300	3.25450300	0.29461400
C	1.58948300	2.27483600	-1.54523600
C	3.70068000	2.05962000	-0.14191200
H	4.11293300	2.02782000	0.87849200
H	3.99100500	1.13429300	-0.65736900
H	4.17401000	2.90492000	-0.66938700
H	1.78777700	1.33586100	-2.08023500
H	2.04837100	3.09468700	-2.12254700
H	0.50180300	2.43587000	-1.52292600
C	-1.77547300	0.63834700	-0.73689300
C	-2.00552500	0.61206400	-2.13680200
C	-2.11014200	1.82671600	-0.04543400
C	-2.57445400	1.70144300	-2.79396200
C	-2.66802700	2.91939200	-0.71580800
C	-2.90924100	2.86804700	-2.09207500
H	-1.73277100	-0.28078200	-2.70602400
H	-1.93609700	1.88611500	1.03044000
H	-2.75117300	1.64281900	-3.87210900
H	-2.92152400	3.82099600	-0.14965300
H	-3.34559400	3.72404800	-2.61291000
C	-1.04251000	-0.45093900	-0.10540500
N	-1.60508200	-1.44201700	0.53217800
C	-0.69848100	-2.50130200	1.08345700
H	0.29968000	-2.07670400	0.93207100
C	-0.82215200	-3.80567400	0.29321300
C	-0.87916200	-2.69175500	2.59198900
H	-0.76674700	-1.73366500	3.12257200
H	-1.84891500	-3.13831100	2.86794500
H	-0.09305600	-3.36922700	2.96184900
H	-1.80456100	-4.29239000	0.42059500
H	-0.05583200	-4.51820200	0.63806500
H	-0.65132100	-3.62960500	-0.77977000
C	-3.07687200	-1.68624300	0.75704400
H	-3.11584900	-2.66642300	1.25139500
C	-3.86669100	-1.82431800	-0.54839700
C	-3.70435300	-0.67292600	1.72019300
H	-3.11538200	-0.58126700	2.64550800
H	-3.79385000	0.32145800	1.26213100
H	-4.71717200	-1.01002600	1.99622700
H	-3.97243400	-0.86601400	-1.07418300
H	-4.87817000	-2.19815000	-0.31910500

H	-3.38714900	-2.54390000	-1.22990100

TS-3a-Z

Sum of electronic and zero-point Energies= -1121.670074
Sum of electronic and thermal Free Energies= -1121.731052

C	1.57937100	1.10711100	-0.49077600
C	2.47708700	2.02373500	0.11695100
C	1.11502900	1.42112900	-1.78594300
C	2.87696700	3.18926400	-0.53335100
C	1.52228400	2.59254700	-2.43521900
C	2.40167400	3.48471200	-1.81873400
H	2.82902200	1.82770000	1.13379300
H	0.43112200	0.72899000	-2.27337100
H	3.55906600	3.88018300	-0.02855900
H	1.14429000	2.80520800	-3.44001100
H	2.71415100	4.40025000	-2.32840900
C	1.14283300	-0.03147200	0.32956100
N	2.00801100	-1.02141400	0.50873600
C	3.23034600	-1.26066600	-0.33314800
H	3.15445300	-0.52232700	-1.14205600
C	3.22435900	-2.65550100	-0.97272400
C	4.54065500	-0.99424900	0.41797000
H	4.57074600	0.02797100	0.82167100
H	4.69990400	-1.70332600	1.24662100
H	5.39052700	-1.10706300	-0.27511000
H	3.34760100	-3.46160700	-0.23097600
H	4.06383600	-2.73760900	-1.68225800
H	2.29395500	-2.83983700	-1.52849400
C	1.88105200	-1.90166800	1.71389900
H	2.65673300	-2.67523200	1.61258400
C	0.53140400	-2.60699200	1.80306400
C	2.17696000	-1.10331100	2.98995300
H	3.16197700	-0.61520400	2.94352600
H	1.41708200	-0.32042000	3.13400100
H	2.16567000	-1.76701600	3.86996500
H	-0.28230300	-1.86971800	1.82225100
H	0.48340400	-3.21019100	2.72385100
H	0.36981000	-3.27855900	0.94847100
C	-1.57937700	1.10710500	0.49078700
C	-2.47709300	2.02372500	-0.11694600
C	-1.11504300	1.42112700	1.78595600
C	-2.87697700	3.18925700	0.53334900
C	-1.52230100	2.59254900	2.43522400
C	-2.40168700	3.48471100	1.81873200
H	-2.82902300	1.82768600	-1.13379000
H	-0.43114100	0.72899100	2.27339500
H	-3.55907300	3.88017300	0.02855000
H	-1.14431100	2.80521400	3.44001700
H	-2.71416600	4.40025200	2.32840100
C	-1.14283200	-0.03147300	-0.32955200
N	-2.00800200	-1.02142200	-0.50873300
C	-3.23033000	-1.26069100	0.33315600
H	-3.15443500	-0.52235900	1.14207100
C	-3.22433400	-2.65553200	0.97271900
C	-4.54064900	-0.99427500	-0.41794600
H	-4.57075300	0.02794800	-0.82163700
H	-4.69990200	-1.70334600	-1.24660000
H	-5.39051200	-1.10710100	0.27514300
H	-3.34761400	-3.46162900	0.23096900
H	-4.06378500	-2.73763700	1.68228400
H	-2.29391100	-2.83988400	1.52845300
C	-1.88103800	-1.90166300	-1.71390300
H	-2.65667300	-2.67526900	-1.61256600
C	-0.53135600	-2.60691600	-1.80312100
C	-2.17702200	-1.10331600	-2.98994700
H	-3.16206000	-0.61525500	-2.94349500
H	-1.41718200	-0.32039100	-3.13401400
H	-2.16572400	-1.76701800	-3.86996100
H	0.28231400	-1.86960500	-1.82243700
H	-0.48340000	-3.21018600	-2.72386300
H	-0.36965700	-3.27840100	-0.94848600

TS-11a

Sum of electronic and zero-point Energies= -560.774066
Sum of electronic and thermal Free Energies= -560.816475

C	-1.21335200	-0.43278600	0.37412700
C	-1.92448800	0.46340400	1.20140800
C	-1.93456800	-1.18994500	-0.57478600
C	-3.30395000	0.62173800	1.05425100
C	-3.31330500	-1.02279700	-0.71568800
C	-4.00636000	-0.11531700	0.09400600
H	-1.38948200	1.03220700	1.96660900
H	-1.40604200	-1.91334900	-1.20163500
H	-3.83539700	1.32722200	1.69942200
H	-3.85207200	-1.61103400	-1.46408300
H	-5.08675500	0.00889100	-0.01560100
C	0.21100400	-0.70563800	0.61144300
N	1.24688900	-0.11341700	0.10845100
C	2.46512000	-0.82960500	0.68756400
H	1.07199500	-1.46344900	1.24104800
C	3.31417000	0.13016700	1.49458800
C	3.24222700	-1.51726400	-0.41603600
H	4.04621900	-2.12222300	0.03543900
H	2.60868300	-2.20478200	-1.00488400
H	3.74379500	-0.82654500	-1.13718000
H	3.82203000	0.92420500	0.89472000
H	4.11859100	-0.43574300	1.99336300
H	2.73312200	0.63564900	2.28668300
C	1.34853200	1.01878600	-0.84909600
H	2.43309800	1.14735300	-0.99525600
C	0.80088300	2.32158900	-0.25659700
C	0.73246900	0.67775800	-2.21027000
H	1.14351400	-0.26383100	-2.60581900
H	-0.36195400	0.58375200	-2.15014700
H	0.96748000	1.47853000	-2.92973400
H	-0.29192000	2.28396100	-0.13702900
H	1.04196600	3.16075000	-0.92868400
H	1.25461900	2.53180000	0.72412800

TS-6a

Sum of electronic and zero-point Energies= -560.765920
Sum of electronic and thermal Free Energies= -560.806531

C	-1.16685400	0.15838700	-0.38532000
C	-1.39986700	0.64619300	0.92153900
C	-2.29534000	-0.14808600	-1.18653400
C	-2.70191800	0.82278400	1.39974200
C	-3.58727800	-0.00121100	-0.69376500
C	-3.80168900	0.49343200	0.60338300
H	-0.53724600	0.89891800	1.54070200
H	-2.13952500	-0.50763300	-2.20861200
H	-2.85656000	1.21318000	2.41020500
H	-4.44129700	-0.25737800	-1.32776000
H	-4.81904600	0.62529600	0.98156800
C	0.18762600	0.13728000	-0.93970500
N	1.27535600	0.01131400	-0.02123200
C	1.45395400	-1.45377900	0.04987600
H	2.50602600	-1.69267200	-0.18060400
C	0.55159500	-1.94662600	-1.11306900
C	1.10149800	-2.07660100	1.39893200
H	1.75745400	-1.68072600	2.19002700
H	0.05951600	-1.85006000	1.67210900
H	1.22049500	-3.17349000	1.36272900
H	-0.39079900	-2.42275700	-0.82039400
H	1.04982600	-2.50936000	-1.91870700
H	0.32466100	-0.86320700	-1.79074500
C	2.48878600	0.77823700	-0.37972000
H	2.93761600	0.35571400	-1.30820400
C	3.52338500	0.67961700	0.74634100
C	2.12959400	2.23863200	-0.65455100
H	1.36878500	2.31335200	-1.44523900
H	1.73235500	2.71893400	0.25530900
H	3.02338900	2.79637000	-0.97745800
H	3.11144300	1.09561700	1.68051900
H	4.42940100	1.24895300	0.48380200
H	3.83505900	-0.35781200	0.94648700

11a

Sum of electronic and zero-point Energies= -560.844725

Sum of electronic and thermal Free Energies= -560.885803

C	1.21780600	-0.49582200	0.12765000
C	1.60352700	0.45931100	-0.84993700
C	2.27418500	-1.17252800	0.80114500
C	2.94650500	0.75259300	-1.09449500
C	3.61030500	-0.88470200	0.54344200
C	3.96519200	0.09198700	-0.39955600
H	0.83867700	0.94329700	-1.45862300
H	2.01950500	-1.93009200	1.54888900
H	3.20002800	1.49398800	-1.85890700
H	4.39044300	-1.42480100	1.08894500
H	5.01548800	0.32023900	-0.59811900
C	-0.13819900	-0.87574800	0.43044400
N	-1.30493500	-0.19122600	0.16581600
C	-2.41384100	-0.86407900	-0.21433100
H	-0.28943800	-1.88901000	0.80221600
C	-3.80047200	-0.31756200	0.00553900
C	-2.26619200	-2.27724900	-0.67791000
H	-3.11930900	-2.56836000	-1.31226400
H	-1.32210700	-2.39905800	-1.23969700
H	-2.22118600	-3.01817000	0.14922500
H	-4.03598700	-0.16003000	1.07823000
H	-4.53884000	-1.03721700	-0.37548500
H	-3.99752900	0.64136400	-0.50415500
C	-1.46670800	1.28508300	0.46195000
H	-2.44099100	1.33627000	0.96593500
C	-1.57021900	2.13758800	-0.80872600
C	-0.45186400	1.82700700	1.46687100
H	-0.39925400	1.19066300	2.36204400
H	0.55873600	1.91334000	1.04788600
H	-0.78026100	2.83256500	1.77663200
H	-0.58443300	2.32266700	-1.25817100
H	-2.01078800	3.11689600	-0.56134400
H	-2.20836500	1.65876200	-1.56645300

5a

Sum of electronic and zero-point Energies= -560.863135

Sum of electronic and thermal Free Energies= -560.904819

C	0.97116000	-0.26308300	0.46721100
C	1.75212100	-1.28480900	-0.09877500
C	1.59552600	0.96524500	0.74931400
C	3.10128300	-1.07570700	-0.40599100
C	2.94185400	1.17936900	0.44269400
C	3.69997900	0.15932700	-0.14288900
H	1.30365800	-2.26167800	-0.29354000
H	1.01694100	1.76327300	1.22238800
H	3.68756700	-1.88563200	-0.84963000
H	3.40302800	2.14544000	0.66715500
H	4.75384400	0.32414400	-0.38373800
C	-0.44827200	-0.51190400	0.91464500
N	-1.65620200	0.15318600	0.45554400
C	-1.50552100	-1.24400400	0.11116100
H	-0.47033600	-0.69533400	2.00307900
C	-1.23822900	-1.69119700	-1.32089900
C	-2.46434900	-2.17556100	0.84440100
H	-2.65700700	-1.80834900	1.86317200
H	-3.43219000	-2.22676100	0.31558900
H	-2.06174600	-3.20121200	0.90532400
H	-2.14793300	-1.56191000	-1.93289500
H	-0.98608400	-2.76459600	-1.34346100
H	-0.41940400	-1.14433900	-1.80274300
C	-1.90522200	1.31901500	-0.39397800
H	-2.90481900	1.12134600	-0.82349300
C	-0.96473500	1.63428600	-1.57573600
C	-2.04570300	2.54714000	0.51889900
H	-2.76682700	2.34550400	1.32479200
H	-1.07989900	2.80310500	0.98636100
H	-2.38358600	3.42838400	-0.05023700
H	0.05674600	1.86594900	-1.24196900
H	-1.34862700	2.51819200	-2.11206800
H	-0.90926000	0.81345500	-2.30323300

6a

Sum of electronic and zero-point Energies= -560.887539

Sum of electronic and thermal Free Energies= -560.928004

C	1.23766100	0.19965700	-0.31368500
C	1.47512800	0.04620200	1.06115300
C	2.32945100	0.15255500	-1.19444400
C	2.77317600	-0.14550000	1.54282600
C	3.63004500	-0.03364000	-0.71506000
C	3.85607600	-0.18317600	0.65707600
H	0.62110500	0.06549600	1.74253300
H	2.15833600	0.26044700	-2.27063400
H	2.94154000	-0.26857400	2.61681400
H	4.46904600	-0.06819100	-1.41610900
H	4.87155900	-0.33361100	1.03426300
C	-0.15520300	0.47271100	-0.83806000
N	-1.30214500	-0.00425900	-0.03744900
C	-2.03395300	1.26968900	-0.24401100
H	-2.75563700	1.18738100	-1.08588300
C	-0.71518200	1.91789100	-0.72212900
C	-2.72392300	1.87612700	0.96982500
H	-3.58545500	1.27471700	1.29908400
H	-2.02010400	1.95153500	1.81416800
H	-3.09273800	2.88894200	0.73305700
H	-0.21578700	2.49787300	0.06789200
H	-0.74791300	2.51048000	-1.64712300
H	-0.21729800	0.12764300	-1.89267200
C	-1.93210700	-1.26777300	-0.41627200
H	-2.23515300	-1.21838200	-1.49142300
C	-3.19773700	-1.51929400	0.40896400
C	-0.94660600	-2.43100300	-0.26655100
H	-0.04005600	-2.28505700	-0.87341200
H	-0.63303300	-2.54244100	0.78404300
H	-1.41853600	-3.37265800	-0.59057700
H	-2.95814000	-1.54385300	1.48479000
H	-3.64740100	-2.48596000	0.13111400
H	-3.96028000	-0.74263800	0.24442000

TS-5a

Sum of electronic and zero-point Energies= -560.836188

Sum of electronic and thermal Free Energies= -560.877388

C	1.28737600	0.07856200	-0.39259500
C	1.60038400	-0.38961600	0.90759400
C	2.36464300	0.40916600	-1.25325900
C	2.92675800	-0.52610900	1.31755100
C	3.68577200	0.26774200	-0.83746900
C	3.97814000	-0.19937800	0.45227500
H	0.77859900	-0.64974900	1.57769100
H	2.14592400	0.77647100	-2.26068400
H	3.14483100	-0.89272100	2.32518000
H	4.49949300	0.52590000	-1.52170900
H	5.01621600	-0.30526500	0.77868700
C	-0.07065300	0.20633000	-0.85877400
N	-1.20917300	-0.07762800	-0.01723100
C	-1.61606500	1.28481600	0.11429800
H	-0.25823100	0.52482200	-1.89325300
C	-2.66849000	1.87495200	-0.77829800
C	-1.12970200	2.05534600	1.30004200
H	-1.85651500	1.97127600	2.13563500
H	-0.17081000	1.66250300	1.66970800
H	-1.01848300	3.12986000	1.07740800
H	-2.71497300	1.36979300	-1.75525300
H	-2.49611100	2.95067000	-0.94769500
H	-3.67550200	1.78597700	-0.32146700
C	-2.15477800	-1.15202000	-0.33646300
H	-2.70469600	-0.91475600	-1.27563200
C	-3.18099000	-1.28619700	0.79199700
C	-1.40001400	-2.46168300	-0.56702100
H	-0.65545600	-2.36032000	-1.37178000
H	-0.87001800	-2.77377900	0.34756000
H	-2.10235200	-3.26090800	-0.85212500
H	-2.68190700	-1.57478800	1.73159100
H	-3.93337700	-2.05272600	0.54604300

H	-3.71671800	-0.34032400	0.97145400

3b_E

Sum of electronic and zero-point Energies= -1040.913322

Sum of electronic and thermal Free Energies= -1040.966326

C	-1.14631600	-1.57185700	-0.18579200
C	-2.05387700	-1.44245000	-1.25583700
C	-1.36269400	-2.61071400	0.74054000
C	-3.14025400	-2.30892300	-1.38703400
C	-2.45342800	-3.47692700	0.61403600
C	-3.34795600	-3.32952500	-0.44992500
H	-1.89138000	-0.65200000	-1.99217400
H	-0.67113000	-2.73464900	1.57812000
H	-3.82817000	-2.19234100	-2.22961800
H	-2.60403600	-4.27098100	1.35116900
H	-4.19915200	-4.00820500	-0.55346000
C	0.05076400	-0.68336300	-0.07333200
C	1.14634900	1.57199800	-0.18573200
C	2.05406200	1.44248800	-1.25565900
C	1.36257300	2.61103100	0.74044400
C	3.14038300	2.30901800	-1.38688600
C	2.45324000	3.47731700	0.61389300
C	3.34789800	3.32981200	-0.44994200
H	1.89170100	0.65191600	-1.99189300
H	0.67093800	2.73504700	1.57795300
H	3.82839600	2.19234200	-2.22937800
H	2.60369100	4.27151200	1.35090600
H	4.19904600	4.00854700	-0.55350600
C	-0.05063400	0.68342600	-0.07333400
C	-2.01865200	1.16864300	1.37668500
C	-1.59621700	2.55194000	-0.59731800
C	-3.53426300	1.29421100	1.19156300
H	-1.68063500	1.95152700	2.09655700
H	-1.76862200	0.19560300	1.82035500
C	-3.10151100	2.70662800	-0.84834300
H	-1.23759900	3.43609000	-0.02381800
H	-1.04766300	2.55501800	-1.55108600
C	-3.89231900	2.59364700	0.46079100
H	-4.02816100	1.25223900	2.17759000
H	-3.89257700	0.42842200	0.60932700
H	-3.29188400	3.67740300	-1.33766200
H	-3.43193200	1.91902900	-1.54807700
H	-4.97706400	2.64647900	0.26702100
H	-3.64717700	3.45675100	1.10824800
C	2.01887300	-1.16857600	1.37660800
C	1.59603500	-2.55218900	-0.59709000
C	3.53444000	-1.29434500	1.19133000
H	1.68086000	-1.95131800	2.09662900
H	1.76898900	-0.19544200	1.82014300
C	3.10128500	-2.70705500	-0.84826700
H	1.23740200	-3.43619700	-0.02338500
H	1.04737200	-2.55539300	-1.55079100
C	3.89227000	-2.59394800	0.46074600
H	4.02844200	-1.25225000	2.17730200
H	3.89281200	-0.42870000	0.60891600
H	3.29150100	-3.67792300	-1.33746600
H	3.43169700	-1.91960100	-1.54817000
H	4.97698300	-2.64693200	0.26685000
H	3.64710500	-3.45691200	1.10838000
N	1.31410200	-1.30326600	0.10161200
N	-1.31411100	1.30315100	0.10155500

3b_Z

Sum of electronic and zero-point Energies= -1040.913998

Sum of electronic and thermal Free Energies= -1040.966753

C	1.37951600	-1.47024500	0.14492400
C	2.26020000	-1.26139800	1.22245300
C	1.70383900	-2.47698100	-0.78765200
C	3.43155300	-2.01250200	1.35283900
C	2.87367100	-3.22838000	-0.65948300
C	3.74490300	-2.99863100	0.41203800
H	2.01715300	-0.49783600	1.96488100
H	1.03719800	-2.65657400	-1.63541600
H	4.10129500	-1.82889300	2.19794500
H	3.11028400	-3.99528700	-1.40281200
H	4.66059200	-3.58771500	0.51379700
C	0.11368600	-0.68807300	0.00372000
C	1.37940800	1.47032800	-0.14488500
C	1.70367200	2.47711400	0.78765200
C	2.26010400	1.26150700	-1.22241400
C	2.87346300	3.22857800	0.65945900
C	3.43141700	2.01266800	-1.35282100
C	3.74471000	2.99884500	-0.41205000
H	1.03703300	2.65669900	1.63541900
H	2.01709500	0.49791500	-1.96482400
H	3.11002600	3.99552000	1.40276600
H	4.10116800	1.82907200	-2.19792400
H	4.66036500	3.58797900	-0.51382600
C	0.11362900	0.68806700	-0.00369000
C	-1.87310500	1.31738800	1.31474500
C	-3.33694700	1.71941600	1.10901300
H	-1.43184400	1.96555100	2.10653000
H	-1.81338300	0.27930100	1.66739400
C	-2.62461700	3.13522800	-0.83927700
C	-3.45636800	3.09610600	0.44699200
H	-3.85586000	1.70528100	2.08280500
H	-3.82870300	0.96436700	0.47231900
H	-2.64433100	4.14303600	-1.28831700
H	-3.05624900	2.44080400	-1.58175200
H	-4.51229900	3.33936300	0.23988900
H	-3.08590300	3.87242600	1.14265700
C	-1.87299800	-1.31752900	-1.31477500
C	-1.17069800	-2.73833100	0.56128100
C	-3.33683300	-1.71956700	-1.10899900
H	-1.43176400	-1.96570800	-2.10656600
H	-1.81329000	-0.27945000	-1.66744400
C	-2.62442800	-3.13534000	0.83929300
H	-0.70412700	-3.53089700	-0.06417900
H	-0.59735100	-2.70719000	1.49957900
C	-3.45621700	-3.09624800	-0.44694900
H	-3.85577200	-1.70546400	-2.08277800
H	-3.82858300	-0.96451200	-0.47230900
H	-2.64412700	-4.14314100	1.28835100
H	-3.05603600	-2.44090300	1.58176900
H	-4.51213800	-3.33951600	-0.23980900
H	-3.08576300	-3.87257600	-1.14261200
N	-1.09541900	-1.42644200	-0.07731300
C	-1.17087700	2.73821700	-0.56132800
H	-0.70427800	3.53077200	0.06412300
H	-0.59756800	2.70708500	-1.49965000
N	-1.09557200	1.42631100	0.07724100

3c_E

Sum of electronic and zero-point Energies= -1735.765611

Sum of electronic and thermal Free Energies= -1735.837700

C	0.48041100	-0.49009600	0.34137200
C	-0.48055700	0.49017000	0.34129900
C	0.47339800	2.27540600	-1.11585900
C	-0.84962600	2.89289600	0.84907800
C	1.39539300	3.48455000	-0.92635100
H	-0.32857500	2.54933000	-1.84217800
H	1.02944500	1.43556500	-1.55351200
C	0.04491700	4.11284200	1.10392800
H	-1.74195700	3.22144500	0.27001100
H	-1.22620300	2.48840800	1.80040000
C	0.66472700	4.62202200	-0.20276300
H	1.76481700	3.82122600	-1.91027700
H	2.27364300	3.17207200	-0.33626800
H	-0.54922900	4.90564300	1.59021300
H	0.84784000	3.82962600	1.80690800
H	1.34983400	5.46432400	-0.00772700
H	-0.13732300	5.01503000	-0.85597600
C	-0.47295200	-2.27551700	-1.11582700
C	0.84946700	-2.89264600	0.84963700
C	-1.39460500	-3.48497100	-0.92671900
H	0.32940100	-2.54910700	-1.84185900

H	-1.02912700	-1.43584300	-1.55365300
C	-0.04470200	-4.11294600	1.10401900
H	1.74219700	-3.22081000	0.27095800
H	1.22546600	-2.48805700	1.80115100
C	-0.66383200	-4.62224600	-0.20294400
H	-1.76361600	-3.82170800	-1.91077800
H	-2.27314300	-3.17279300	-0.33689700
H	0.54955600	-4.90556800	1.59045600
H	-0.84800400	-3.83012100	1.80672300
H	-1.34873500	-5.46478500	-0.00821600
H	0.13858200	-5.01495700	-0.85588500
N	0.11034700	-1.84545400	0.15419200
N	-0.11062500	1.84548200	0.15386200
C	1.93572600	-0.18150600	0.46622000
C	2.86777300	-0.72067700	-0.44838900
C	2.42181900	0.59583900	1.54031300
C	4.22851400	-0.47028800	-0.29619800
H	2.50718500	-1.32754800	-1.28382800
C	3.78445600	0.84675200	1.70340500
H	1.70529400	0.99716700	2.26028000
C	5.39285900	-0.94432300	-1.16947300
C	4.69339100	0.31468300	0.77933900
H	4.13289600	1.44611500	2.54942700
C	6.58422700	-0.32080600	-0.43866600
C	6.15595900	0.40782500	0.69354300
C	7.93932200	-0.39433400	-0.75301300
C	7.08430300	1.06312200	1.51059200
C	8.87018200	0.26217700	0.06665800
H	8.28342500	-0.95564800	-1.62706200
C	8.44417400	0.98493900	1.18914800
H	6.75661800	1.62865000	2.38743500
H	9.93603900	0.20937300	-0.17231700
H	9.18077700	1.49182600	1.81887000
C	-1.93586700	0.18157600	0.46631700
C	-2.86792900	0.72077000	-0.44830000
C	-2.42196900	-0.59579600	1.54035200
C	-4.22865500	0.47035900	-0.29616900
H	-2.50731000	1.32767000	-1.28370700
C	-3.78461900	-0.84672800	1.70340000
H	-1.70549700	-0.99717100	2.26034400
C	-5.39299700	0.94440800	-1.16943700
C	-4.69354000	-0.31466900	0.77933900
H	-4.13304800	-1.44612000	2.54940700
C	-6.58436700	0.32084200	-0.43866700
C	-6.15610400	-0.40781900	0.69352500
C	-7.93945700	0.39435200	-0.75304000
C	-7.08445100	-1.06316800	1.51052900
C	-8.87032000	-0.26220500	0.06659300
H	-8.28355800	0.95569200	-1.62707200
C	-8.44431800	-0.98500100	1.18906300
H	-6.75677100	-1.62873000	2.38735200
H	-9.93617300	-0.20940900	-0.17240200
H	-9.18092000	-1.49193100	1.81875100
C	5.49017000	-2.48565300	-1.18891700
H	5.58077800	-2.89055300	-0.16912500
H	4.59455800	-2.92804500	-1.65527900
H	6.36861300	-2.81541900	-1.76770400
C	5.27264500	-0.40328400	-2.61116400
H	4.37498700	-0.80797700	-3.10700600
H	5.20221800	0.69542400	-2.61846400
H	6.14931000	-0.69439900	-3.21289400
C	-5.49033300	2.48574200	-1.18878900
H	-5.58097300	2.89056700	-0.16897000
H	-4.59471200	2.92817800	-1.65509600
H	-6.36876200	2.81553300	-1.76758300
C	-5.27278800	0.40343700	-2.61115000
H	-4.37509800	0.80809500	-3.10696300
H	-5.20242200	-0.69527400	-2.61849700
H	-6.14942700	0.69462600	-3.21288100

3c_Z

Sum of electronic and zero-point Energies= -1735.766230
Sum of electronic and thermal Free Energies= -1735.837298

C	0.68333500	-1.82394900	0.08662600
C	-0.68354000	-1.82388900	-0.08661200
C	-1.47116600	-3.80368400	1.15906500
C	-2.63910300	-3.12538500	-0.89300600
C	-1.83212100	-5.27203500	0.91291100
H	-2.22009200	-3.36326600	1.85702600
H	-0.48840100	-3.73240600	1.64340900
C	-2.98182800	-4.58454100	-1.21209900
H	-3.51296300	-2.66637200	-0.38018600
H	-2.48913100	-2.55406800	-1.82096100
C	-3.10752400	-5.40715100	0.07428500
H	-1.94373600	-5.78482800	1.88363800
H	-0.99580700	-5.76129900	0.38546000
H	-3.92036700	-4.61739000	-1.79155300
H	-2.19019600	-5.01377300	-1.85151700
H	-3.31296900	-6.46653200	-0.15486400
H	-3.97236300	-5.03895500	0.65775700
C	1.47072900	-3.80381500	-1.15900700
C	2.63879800	-3.12556800	0.89297200
C	1.83158400	-5.27219600	-0.91287600
H	2.21972100	-3.36339800	-1.85689100
H	0.48798200	-3.73239400	-1.64338600
C	2.98154100	-4.58472800	1.21200700
H	3.51256700	-2.66653500	0.38002000
H	2.48896000	-2.55425800	1.82095700
C	3.10706100	-5.40736500	-0.07437300
H	1.94303900	-5.78502500	-1.88360400
H	0.99527700	-5.76135500	-0.38532400
H	3.92015200	-4.61760200	1.79134700
H	2.18997300	-5.01391600	1.85152900
H	3.31247400	-6.46674800	0.15480000
H	3.97186500	-5.03924500	-0.65794600
N	1.42407900	-3.03413000	0.08657200
N	-1.42449500	-3.03395000	-0.08646900
C	1.44204900	-0.56217500	0.33236700
C	2.57413400	-0.24706400	-0.45437600
C	1.08074200	0.32044100	1.37124400
C	3.29266900	0.91807100	-0.21493900
H	2.86363000	-0.91995000	-1.26659800
C	1.79831500	1.49296300	1.61934600
H	0.21898800	0.07292800	1.99481200
C	4.52471000	1.45139300	-0.95083200
C	2.90790100	1.79499100	0.82331200
H	1.49262500	2.15877400	2.43138500
C	4.79070400	2.74828000	-0.18275300
C	3.83934200	2.92969700	0.84585400
C	5.79670900	3.69137700	-0.38100300
C	3.89532500	4.05520200	1.67573200
C	5.85327800	4.81932800	0.45175100
H	6.53919800	3.56225900	-1.17437900
C	4.90902800	4.99825800	1.47174000
H	3.16035100	4.19958600	2.47252200
H	6.63998600	5.56440900	0.30360500
H	4.96493300	5.88242100	2.11300400
C	-1.44207500	-0.56202400	-0.33240600
C	-2.57429300	-0.24695300	0.45418000
C	-1.08048700	0.32075000	-1.37104600
C	-3.29266500	0.91829300	0.21483900
H	-2.86399600	-0.91998500	1.26620400
C	-1.79790500	1.49339000	-1.61905500
H	-0.21867600	0.07327200	-1.99454000
C	-4.52477400	1.45162200	0.95061100
C	-2.90759300	1.79538000	-0.82316300
H	-1.49200900	2.15932400	-2.43091500
C	-4.79051900	2.74865000	0.18268500
C	-3.83893000	2.93017100	-0.84569200
C	-5.79650000	3.69178600	0.38087700
C	-3.89463500	4.05583300	-1.67537700
C	-5.85279900	4.81988800	-0.45169000
H	-6.53918100	3.56258600	1.17406100
C	-4.90831000	4.99893200	-1.47143900

H	-3.15946700	4.20031000	-2.47197100
H	-6.63948800	5.56499800	-0.30358900
H	-4.96400700	5.88321700	-2.11255200
C	5.71580100	0.47523000	-0.83522700
H	5.95639100	0.26526600	0.21842100
H	5.48605600	-0.48185000	-1.33177600
H	6.61500100	0.89573800	-1.31486000
C	4.21363200	1.73323200	-2.43724000
H	3.95687400	0.80074900	-2.96629600
H	3.36710200	2.42954200	-2.54032300
H	5.08677500	2.17739700	-2.94293900
C	-5.71593000	0.47559100	0.83477800
H	-5.95641700	0.26572300	-0.21891400
H	-5.48633800	-0.48155100	1.33128100
H	-6.61515200	0.89613900	1.31433100
C	-4.21382500	1.73327200	2.43710100
H	-3.95722800	0.80069900	2.96607400
H	-3.36722800	2.42947300	2.54034400
H	-5.08697700	2.17747900	2.94274900

TS_Im-Prop

Sum of electronic and zero-point Energies= -560.806481

Sum of electronic and thermal Free Energies= -560.848779

6	1.336268000	-0.464077000	0.090955000
6	2.057507000	-0.759311000	-1.086396000
6	2.040938000	0.067727000	1.193676000
6	3.427299000	-0.502740000	-1.164188000
6	3.415128000	0.305694000	1.111612000
6	4.115866000	0.028527000	-0.066958000
1	1.532846000	-1.195833000	-1.940733000
1	1.505770000	0.280425000	2.123387000
1	3.965162000	-0.729666000	-2.089365000
1	3.942566000	0.714073000	1.978745000
1	5.190843000	0.217389000	-0.128032000
6	-0.090109000	-0.764880000	0.183776000
7	-1.062609000	0.029084000	0.177809000
6	-1.021333000	1.494679000	0.054527000
1	-0.026172000	1.819923000	0.406573000
6	-2.073620000	2.155599000	0.949897000
6	-1.149652000	1.921762000	-1.414161000
1	-0.353835000	1.467398000	-2.024077000
1	-2.121016000	1.626934000	-1.839344000
1	-1.058273000	3.016973000	-1.496788000
1	-3.098544000	1.927947000	0.617816000
1	-1.949139000	3.250015000	0.926117000
1	-1.969946000	1.826159000	1.995802000
6	-2.740740000	-1.052965000	0.427535000
1	-3.062739000	-0.519285000	1.327596000
6	-2.148206000	-2.339387000	0.602343000
6	-3.556442000	-0.756414000	-0.806727000
1	-3.750697000	0.317830000	-0.947637000
1	-3.060311000	-1.139726000	-1.713557000
1	-4.539281000	-1.257141000	-0.736012000
1	-0.886980000	-1.988057000	0.394171000
1	-2.358153000	-3.089123000	-0.172453000
1	-2.141270000	-2.755111000	1.617773000

Propene

Sum of electronic and zero-point Energies= -117.743930

Sum of electronic and thermal Free Energies= -117.768950

6	0.132054000	0.452012000	0.000056000
1	0.164308000	1.549887000	0.000088000
6	1.286916000	-0.219758000	-0.000072000
6	-1.236418000	-0.162824000	-0.000016000
1	-1.816591000	0.157276000	-0.883507000
1	-1.190605000	-1.262967000	-0.000482000
1	-1.816429000	0.156547000	0.883842000
1	2.250373000	0.297747000	-0.000055000
1	1.313633000	-1.315069000	0.000303000

Imine

Sum of electronic and zero-point Energies= -443.103051

Sum of electronic and thermal Free Energies= -443.139758

6	0.782089000	0.467276000	0.357342000
6	1.898983000	1.266317000	0.047724000
6	0.927617000	-0.931827000	0.303696000
6	3.108007000	0.692596000	-0.347882000
6	2.142296000	-1.507144000	-0.077762000
6	3.232303000	-0.698838000	-0.414246000
1	1.809557000	2.354652000	0.112996000
1	0.097741000	-1.578237000	0.591680000
1	3.958919000	1.331965000	-0.596702000
1	2.239428000	-2.595517000	-0.105741000
1	4.180111000	-1.152396000	-0.715151000
6	-0.468544000	1.163039000	0.782403000
7	-1.696243000	0.913079000	0.558836000
6	-2.175844000	-0.182716000	-0.270688000
1	-1.361199000	-0.677798000	-0.833407000
6	-3.165079000	0.399857000	-1.288382000
6	-2.855613000	-1.224856000	0.627691000
1	-2.144601000	-1.665806000	1.344549000
1	-3.666070000	-0.756312000	1.208111000
1	-3.283802000	-2.039578000	0.022233000
1	-3.996206000	0.903220000	-0.769721000
1	-3.576674000	-0.395078000	-1.931134000
1	-2.670410000	1.142012000	-1.934059000
1	-0.270390000	2.075097000	1.375112000

Supplementary References

[1] Gaussian 09, Revision **D.01**, Frisch, M. J., Trucks, G. W., Schlegel, H. B., Scuseria, G. E., Robb, M. A., Cheeseman, J. R., Scalmani, G., Barone, V., Mennucci, B., Petersson, G. A., Nakatsuji, H., Caricato, M., Li, X., Hratchian, H. P., Izmaylov, A. F., Bloino, J., Zheng, G., Sonnenberg, J. L., Hada, M., Ehara, M., Toyota, K., Fukuda, R., Hasegawa, J., Ishida, M., Nakajima, T., Honda, Y., Kitao, O., Nakai, H., Vreven, T., Montgomery, J. A., Jr., Peralta, J. E., Ogliaro, F., Bearpark, M., Heyd, J. J., Brothers, E., Kudin, K. N., Staroverov, V. N., Kobayashi, R., Normand, J., Raghavachari, K., Rendell, A., Burant, J. C., Iyengar, S. S., Tomasi, J., Cossi, M., Rega, N., Millam, J. M., Klene, M., Knox, J. E., Cross, J. B., Bakken, V., Adamo, C., Jaramillo, J., Gomperts, R., Stratmann, R. E., Yazyev, O., Austin, A. J., Cammi, R., Pomelli, C., Ochterski, J. W., Martin, R. L., Morokuma, K., Zakrzewski, V. G., Voth, G. A., Salvador, P., Dannenberg, J. J., Dapprich, S., Daniels, A. D., Farkas, Ö., Foresman, J. B., Ortiz, J. V., Cioslowski, J., Fox, D. J. Gaussian, Inc., Wallingford CT, (2009).

[2] Becke, A. D. Density-Functional thermochemistry. III. The role of excat exchange. *J. Chem. Phys.* **98**, 5648-5652 (1993).

[3] Becke, A. D. Density-functional exchange-energy approximation with correct asymptotic behavior. *Phys. Rev. A.*, **38**, 3098-3100 (1988).

[4] Lee, C., Yang, W., Parr, R. G. Development of the Colle-Salvetti correlation energy formula into a functional of the electron density. *Phys. Rev.*, **B 37**, 785-789 (1988).

[5] Weigend, F., Ahlrichs, R. Balanced basis sets of split valences, triple zeta valence and quadruple zeta valence quality for H to Rn: Design and assessment of accuracy. *Phys. Chem. Chem. Phys.*, **7**, 3297-3305 (2005).

[6] Fukui, K. The path of chemical reactions – the IRC approach. *Acc. Chem. Res.*, **14**, 363-368 (1981).

[7] Hratchian, H. P., Schlegel, H.B., in *Theory and Applications of Computational Chemistry: The First 40 Years*, Ed. Dykstra, C. E., Frenking, G., Kim, K. S., Scuseria, G. Elsevier, Amsterdam, 195 (2005).

[8] GaussView, Version 5, Dennington, R., Keith, T., Millam, J., *Semichem Inc.*, Shawnee Mission, KS, (2009).
